# Glutamine: fructose-6-phosphate amidotransferase (GFAT) in the pathology of diseases: a review

**DOI:** 10.1038/s41420-025-02898-8

**Published:** 2025-12-14

**Authors:** Caiting Yang, Fengyu Chu, Xiaoli Chen, Fanqi Meng, Yinhao Li, Jingwen Chen, Chaoyang Sun, Yahui Shang, Ruibin Guo, Jianming Wang, Changxin Wu, Huiping Duan, Miaomiao Shao, Wei Yuan

**Affiliations:** 1 Shanxi Provincial Key Laboratory for Medical Molecular Cell Biology, Key Laboratory of Chemical Biology and Molecular Engineering of Ministry of Education and Institute of Biomedical Sciences, Shanxi University, Taiyuan, Shanxi China; 2 Department of Oncology, General Hospital of Yangquan Coal Industry Group; Department of Oncology and Interventional Radiology, Yang Quan Hospital of Shanxi Medical University, Yangquan, Shanxi China; 3 Tuberculosis Department, Fourth People’s Hospital of Taiyuan, Taiyuan, Shanxi China; 4 School of Medicine, Nanjing University of Chinese Medicine, Nanjing, Jiangsu China

**Keywords:** Cancer, Cardiovascular diseases, Metabolic disorders, Prognostic markers, Cell signalling

## Abstract

Glutamine: fructose-6-phosphate amidotransferase (GFAT), a conserved enzyme across prokaryotic and eukaryotic species, is the first and rate-limiting step in the hexosamine biosynthetic pathway (HBP), diverting 2–5% of fructose-6-phosphate derived from glucose toward the synthesis of uridine diphosphate N-acetylglucosamine (UDP-GlcNAc), a key substrate for the glycosylation of proteins and lipids. While substantial progress has been made in elucidating the basic biochemical properties and regulatory mechanisms of GFAT, its functional impact on pathological processes remains incompletely understood. Emerging evidence implicates GFAT in a spectrum of human diseases, including cancer, diabetes, cardiovascular disorders, and neurodegenerative conditions such as Alzheimer’s disease. This review aims to provide a comprehensive synthesis of current insights into GFAT’s role in disease etiology, with the goal of informing future research and therapeutic strategies targeting this essential metabolic regulator.

## Facts

- Glutamine: fructose-6-phosphate amidotransferase (GFAT) is the first and rate-limiting enzyme of the hexosamine biosynthetic pathway, diverting 2–5% of glucose-derived fructose-6-phosphate to UDP-GlcNAc, a key substrate for multiple glycosylation processes.
- GFAT drives cancer progression by enhancing O-GlcNAcylation/N-glycosylation, stabilizing oncogenic proteins (β-catenin, PD-L1), remodeling the ECM via hyaluronan, and activating PI3K/AKT, ERK/MAPK, and Wnt/β-catenin pathways.
- GFAT isoforms act differently in the heart: GFAT1 activates mTOR and drives early hypertrophy, whereas GFAT2 mainly signals via Akt.
- In diabetes, chronic GFAT activation under hyperglycemia leads to excessive UDP-GlcNAc production, O-GlcNAcylation of insulin signaling proteins, impaired GLUT4 trafficking, and insulin resistance.
- GFAT’s isoform-specific roles represent targets for precise, tissue-specific therapy.

## Open questions

- How do GFAT1 and GFAT2 differentially regulate disease progression across tissues?
- What are the molecular mechanisms linking GFAT-driven O-GlcNAcylation with other post-translational modifications such as phosphorylation, acetylation, ubiquitination, and lactylation?
- How does GFAT integrate with other metabolic pathways, including the pentose phosphate pathway, lipid metabolism, and amino acid metabolism?
- Under what conditions does GFAT confer protective effects, and how can these be separated from its pathogenic roles?
- What strategies can be used to design selective, isoform- and tissue-specific GFAT modulators with therapeutic potential?

## Introduction

**Fig. 1:**
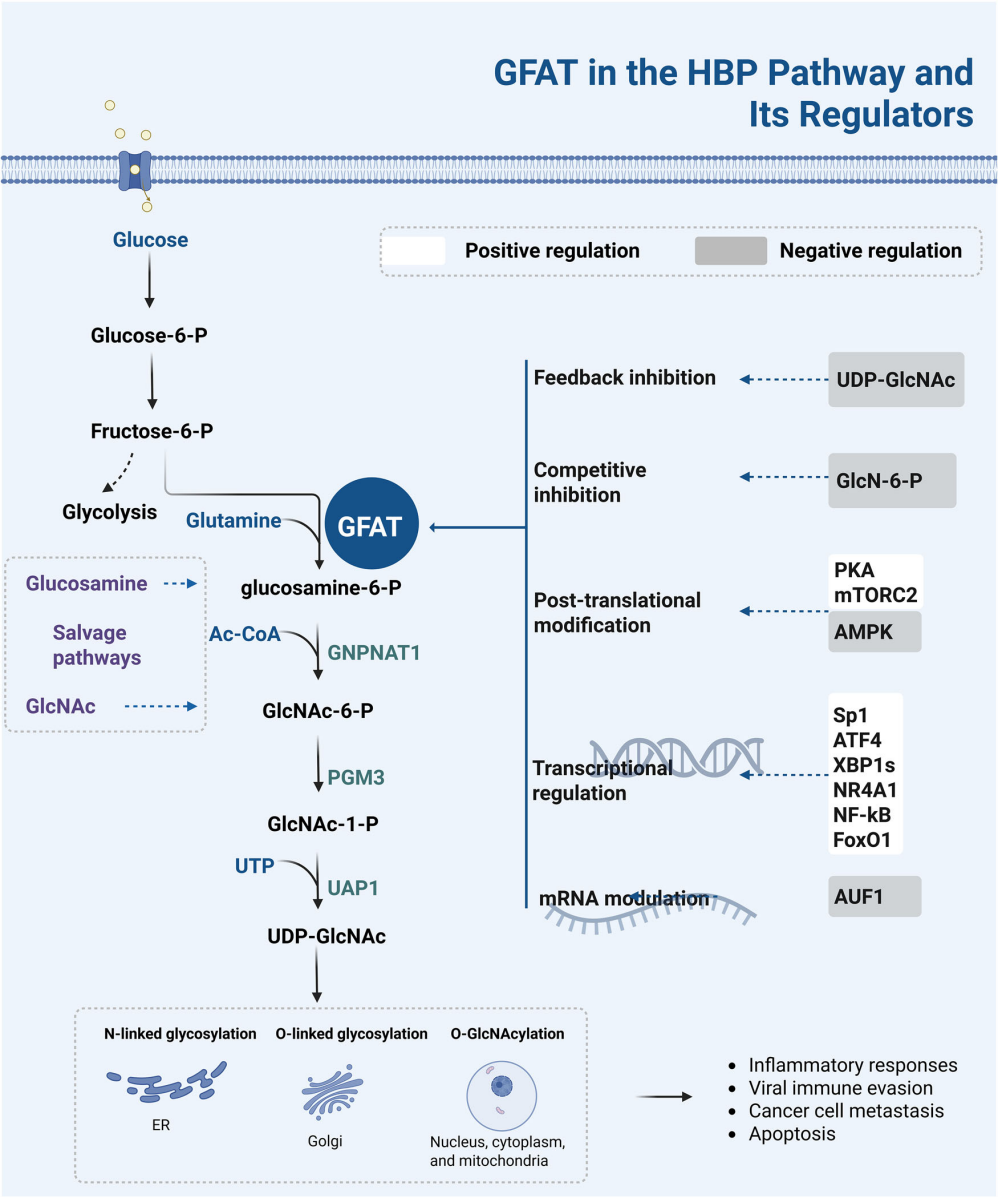
GFAT in the hexosamine biosynthetic pathway (HBP) and its regulators. GFAT, encoded by the *GFPT* gene, is the first and rate-limiting enzyme in the HBP. GFAT regulation occurs at multiple levels, such as allosteric control by metabolites, post-translational modifications, and modulation of mRNA and protein expression, ensuring close coordination with other metabolic processes and enabling effective cellular responses to fluctuations in nutrient levels and other external or internal signals.

Glutamine: fructose-6-phosphate amidotransferase (GFAT), encoded by the *GFPT* gene, is the first and rate-limiting enzyme in the hexosamine biosynthetic pathway (HBP). This enzyme can initiate a distinct branch of glucose metabolism [1], converting approximately 2–5% of the fructose-6-phosphate derived from glucose to glucosamine-6-phosphate with glutamine as the nitrogen donor [2]. Through subsequent enzymatic steps involving acetylcoenzyme A (Ac-CoA) and uridine-5’-triphosphate (UTP), glucosamine-6-phosphate undergoes further metabolism into uridine diphosphate N-acetylglucosamine (UDP-GlcNAc). As an essential precursor for glycosylation, UDP-GlcNAc plays a crucial role in cell signaling, protein stability, and metabolic regulation by modifying proteins and lipids. UDP-GlcNAc serves as a key substrate for N-glycosylation in eukaryotic cells. This process initiates in the endoplasmic reticulum (ER) and undergoes further processing and termination in the Golgi apparatus [3]. Additionally, UDP-GlcNAc is instrumental in O-glycosylation, occurring primarily in the Golgi apparatus, and O-GlcNAcylation, characterized by the addition of a single GlcNAc to serine or threonine residues within mainly nuclear, cytoplasmic, and mitochondrial proteins, as well as S-glycosylation [4]. Disruptions in glycosylation can profoundly impact cellular processes, influencing inflammation, viral immune evasion, cancer metastasis, and apoptosis [5] (Fig. 1).

As the HBP integrates metabolites from carbohydrates, fatty acids, amino acids, and nucleotides [6], it functions as a general “sensor” of energy availability [4, 7–9], essential for cellular homeostasis [10]. Increasing evidence links the HBP to protein quality control, homeostasis, proteotoxic diseases [11–13], aging and age-related diseases, including neurodegenerative diseases, with its elevated activation potentially serving as a cancer biomarker [14]. In addition, the HBP has been implicated in the fusion of circadian and metabolic signals [15]. Since the HBP is pivotal for cellular signaling, cell growth, stress response, and diverse physiological and pathological processes, a comprehensive investigation of GFAT, a key enzyme in this pathway, remains imperative.

The ubiquitous presence of GFAT across all biological species, spanning both prokaryotic and eukaryotic domains, underscores its essential role in cellular function. Structurally and functionally distinct, this enzyme contains a 27 kDa glutaminase domain at the N-terminus that hydrolyzes glutamine into glutamate and ammonia, and a 40 kDa isomerase domain at the C-terminus that uses ammonia to convert fructose-6-phosphate into glucosamine-6-phosphate (GlcN-6-P) [16], the rate-limiting step in hexosamine biosynthesis. Extensive studies on *Escherichia coli* GlmS, the bacterial GFAT homolog, reveal a functional dimer with glutaminase and isomerase domains linked by a flexible connector, with a solvent-inaccessible hydrophobic channel facilitating ammonia transfer between the two domains [17–20]. The fungal counterpart of GFAT, *Candida albicans* Gfa, exists as a tetramer composed of two tightly bound dimers, resembling the structural configuration of *E. coli* GlmS [20, 21]. Similar to human GFAT1 (hGFAT1), hGFAT2 can form tetramers and higher-order oligomers [22, 23] though the potential formation of GFAT1–GFAT2 hetero-oligomers remains uncertain.

**Fig. 2:**
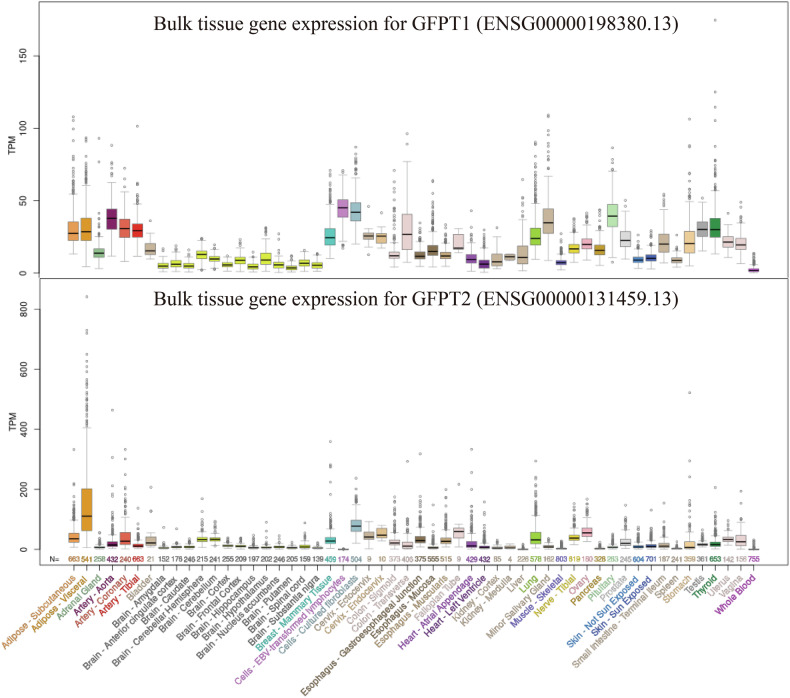
Expression of the two GFAT isoform genes, *GFPT1* and *GFPT2*, across human tissues. *GFPT1* shows broad, moderate expression, whereas *GFPT2* is highly enriched in neural tissues. Sample numbers for each tissueare shown below the plots.

Two distinct isoforms, GFAT1 and GFAT2, (Table 1), share approximately 75.0% amino acid sequence homology in both humans and mice [24]. Functional differences include the slower GlcN-6-P synthesis rate of GFAT2 compared to GFAT1, while their primary divergence lies in tissue-specific expression patterns [24] (Fig. 2). Cell type-dependent expression preferences further distinguish these isoforms; for example, GFAT2 predominates in cardiac fibroblasts, whereas GFAT1 serves as the principal isoform in cardiac myocytes [25]. The essential roles of both isoforms are underscored by the lethality of individual gene deletions, with neither capable of compensating for the other’s absence. Notably, dietary supplementation with ᴅ-glucosamine-6-phosphate rescues the viability of *gfat2*^*−/−*^ mutants but fails to restore survival in *gfat1*^*−/−*^ mutants [26].

**Table 1:** Differences between GFAT1 and GFAT2 isoforms in humans.

Feature	GFAT1	GFAT2
Length	699 amino acids	682 amino acids
Mass	78 kDa	77 kDa
Chromosomal location	2p13.3	5q35.3
Subcellular location	cytosol	cytosol
Tissue specificity	Ubiquitously expressed; higher in muscle, heart, placenta, pancreas, and testis [24, 25, 27, 28]	Predominantly in the central nervous system, especially the spinal cord [24]
Function	Regulates the entry of glucose into the hexosamine biosynthesis pathway. Important for controlling the supply of substrates for N- and O-linked protein glycosylation. Influences the circadian rhythms of clock genes BMAL1 and CRY1. Helps manage the metabolic changes of cytosolic UDP-GlcNAc and its role in hyaluronan production during tissue remodeling [29].	Manages the flow of glucose into the hexosamine pathway. Likely involved in maintaining the supply of substrates necessary for N- and O-linked protein glycosylation.

Notably, although GFAT’s expression patterns and isoform specificity are crucial for tissue-dependent metabolic regulation, the enzyme’s activity is also finely tuned by multiple regulatory mechanisms to maintain metabolic balance. Increased expression does not necessarily equate to enhanced enzymatic activity, as GFAT is subject to multilayered regulation. Post-translational mechanisms, including phosphorylation, feedback inhibition by UDP-GlcNAc [27], competitive inhibition by GlcN-6-P [28], and signaling cues such as PKA [29], mTORC2 [30] or AMPK [31], can modulate GFAT catalytic function independent of expression levels. Additionally, enhanced GFAT enzyme activity is not the only way to increase HBP flux. Glucosamine and GlcNc can also bypass GFAT through the salvage pathway to increase the production of UDP-GlcNAc [32].

## GFAT in cancer

**Fig. 3:**
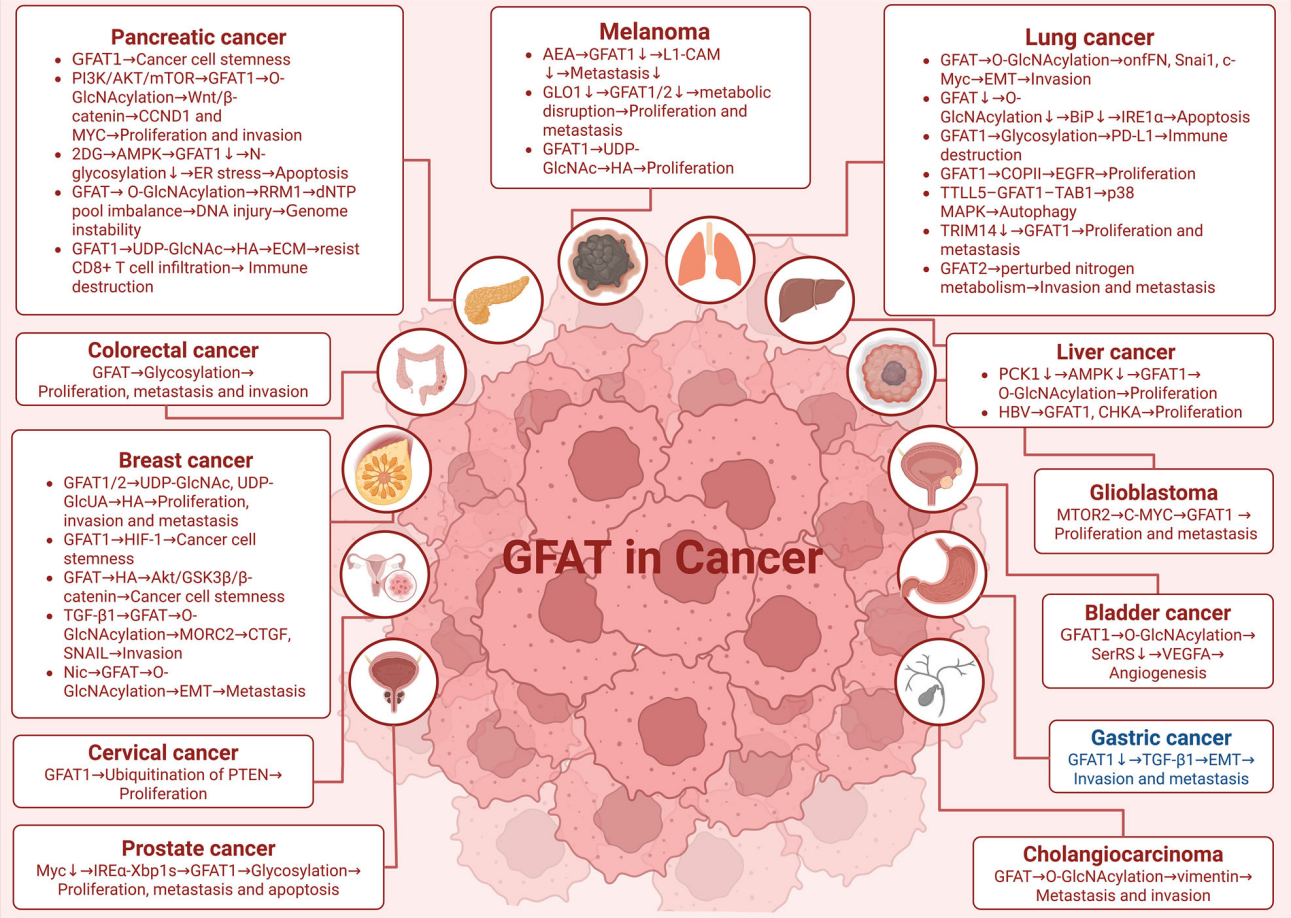
Overview of GFAT-mediated functions and associated signaling pathways in diverse cancer types. GFAT plays a key role in cancer by driving metabolic reprogramming, enhancing O-GlcNAcylation, and promoting oncogenic signaling. Its overexpression in cancers such as pancreatic, lung, breast, and liver cancer is linked to increased proliferation, invasion, immune evasion, and poor prognosis. Mechanistically, GFAT promotes cancer progression by boosting UDP-GlcNAc production, stabilizing oncogenic proteins (e.g., β-catenin, PD-L1), remodeling the ECM through increased hyaluronan synthesis, and activating pro-tumor pathways such as PI3K/AKT, ERK/MAPK, and Wnt/β-catenin. It also impairs DNA repair by driving genomic instability, accelerating tumorigenesis. However, GFAT functions as a tumor suppressor in gastric cancer by inhibiting EMT. This dual role highlights its complex, tissue-specific functions.

Cellular metabolic reprogramming is a core hallmark of cancer. The Warburg effect reflects the preferential reliance of cancer cells on glycolysis for energy production, even under aerobic conditions [33–35]. Within this metabolic landscape, the HBP, branching from glycolysis at fructose-6-phosphate, plays a crucial role in cancer progression. As the rate-limiting enzyme of HBP, GFAT has been shown to be highly expressed in most cancers, a trait closely linked to increased tumor malignancy and poor prognosis. This chapter explores the diverse roles of GFAT in various cancers, including pancreatic, lung, breast, liver, gastric, melanoma, glioblastoma, prostate, bladder, colorectal, and cholangiocarcinoma (Fig. 3).

### GFAT in pancreatic cancer

Pancreatic cancer (PC) remains one of the most lethal malignancies, with a critically low 5-year survival rate of only 13% [36]. GFAT1 has been shown to be overexpressed in pancreatic cancer [37, 38], correlating with increased cell proliferation, invasion, therapeutic resistance, and stemness maintenance [38–40]. High GFAT1 expression has been linked to lymph node metastasis, advanced pTNM stage, and reduced overall survival (OS) [37], establishing GFAT1 as an independent prognostic marker for adverse clinical outcomes. Targeting GFAT1 can disrupt the tumor microenvironment and enhance immune responses, making pancreatic ductal adenocarcinoma (PDAC), the predominant pathological type of pancreatic cancer (>90%) [41] with a 12% 5-year survival rate [42], more responsive to immune checkpoint inhibitor therapy [43], thus leading to tumor regression and extended survival.

Mechanistically, GFAT1 can influence the hyaluronan synthesis by regulating the production of UDP-GlcNAc. GFAT1 inhibition can cause remodeling of the extracellular matrix (ECM) by reducing hyaluronan and collagen, thereby promoting CD8 + T cell infiltration and sensitizing the tumors to anti-PD1 immunotherapy [43]. Notably, Cancer cells can sustain the fidelity of glycosylation precursor pools by acquiring nutrients from the tumor microenvironment [44]. HA can circumvent GFAT1 to replenish the HBP through the GlcNAc salvage pathway, further complicating therapeutic strategies.

Stimulation of the PI3K/AKT/mTOR signaling pathway transcriptionally upregulates *GFPT1* expression, thereby increasing HBP flux and promoting β-catenin O-GlcNAcylation, which enhances Wnt/β-catenin signaling to drive PDAC cell proliferation and invasion [38]. Metabolic stress or pharmacological inhibition can suppress GFAT1 activity post-translationally. The glycolytic inhibitor 2-deoxy-D-glucose (2DG), through AMPK activation and subsequent phosphorylation-mediated inactivation of GFAT1 disrupts protein N-glycosylation, triggering ER stress, ultimately culminating in apoptosis. The co-administration of 2DG and metformin further potentiates these effects, highlighting a possible therapeutic approach for PDAC [39]. Moreover, GFAT can induce genomic instability by inducing O-GlcNAcylation [45]. A significant link to pancreatic cancer progression emerges through RRM1 (Ribonucleotide Reductase M1))-O-GlcNAcylation-mediated dNTP pool imbalance, leading to DNA damage and tumorigenesis.

In conclusion, GFAT emerges as a critical molecular target in pancreatic cancer, with profound implications for tumor progression, immune evasion, and genomic instability. Targeting GFAT offers a multifaceted approach to combat pancreatic cancer by simultaneously disrupting cancer cell metabolism, enhancing the efficacy of existing therapies like immune checkpoint inhibitors, and diminishing the resilience of cancer stem cells. However, the compensatory mechanisms within the tumor microenvironment, such as nutrient scavenging and metabolic bypass pathways like the GlcNAc salvage route, highlight the need for combinatorial therapeutic strategies.

### GFAT in lung cancer

Lung cancer, the foremost cause of cancer-related mortality worldwide [46], predominantly manifests as non-small-cell lung carcinoma (NSCLC), comprising approximately 85% of cases, with lung adenocarcinoma (LUAD) and lung squamous-cell carcinoma (LUSC) representing the most prevalent subtypes [47]. GFAT expression has been found to be elevated in both NSCLC cell lines and tumor tissues [48]. It plays a diverse role in lung cancer, specifically in the regulation of cell proliferation, metastasis, apoptosis, autophagy, chemoresistance, immune evasion, and senescence.

Despite the high glucose consumption of LUAD cells, poor vascular supply often leads to low glucose availability in parts of the tumor microenvironment. According to TCGA data, immunoblotting, and immunohistochemical analysis of LUAD tissue microarrays, GFAT1 was highly expressed at both the mRNA and protein levels. Consistently, LUAD samples with high GFAT1 expression exhibited elevated levels of HBP intermediates (GlcNAc-6-P and UDP-HexNAc). Notably, high GFAT1 protein expression correlates with the epidermal growth factor receptor (EGFR) activation upregulated by coat complex II (COPII) in LUAD patient samples, distinguishing it from other lung cancer subtypes and highlighting its role in promoting tumor progression under nutrient stress [49]. GFAT1 can also form a TTLL5-GFAT1-TAB1 complex upon glucose deprivation, promoting transforming growth factor β-activated kinase 1 binding protein 1 (TAB1) glutamylation, activating P38 mitogen-activated protein kinase (MAPK) signaling, and driving autophagy to help cancer cells survive [50]. The oncoprotein Tripartite Motif 14 (TRIM14), a member of the E3 ligase TRIM family, can suppress NSCLC cell proliferation and migration through ubiquitination and degradation of GFAT1 [51].

Under high glucose conditions, elevated GFAT protein expression increases HBP activity, leading to the production of UDP-GlcNAc, which fuels the O-glycosylation of onfFN, specifically in the hexapeptide (VTHPGY) contained by its variable region (V or IIICS domain). This glycosylation promotes EMT and facilitates A549 cell morphology changes, migration, and invasion [52]. Reciprocally, EMT can reinforce HBP flux by enhancing GFAT protein expression, as shown by transcriptomic and metabolic analyses, leading to KrasG12D-induced lung tumorigenesis. Elevated O-GlcNAcylation posttranslational modification stabilizes oncoproteins like Snail and c-Myc, while suppressing KRASG12D-induced senescence (OIS) or apoptosis (OIA), a tumor-suppressive mechanism, thus driving more aggressive cancer growth [53].

GFAT can regulate the expression of the endoplasmic reticulum (ER) chaperone binding immunoglobulin protein (BiP), which is associated with cisplatin resistance. Reducing BiP levels via pharmacological inhibition of GFAT activity or knockdown of GFAT activates inositol-requiring enzyme 1α (IRE1α), thereby increasing cisplatin-induced cell death [48]. Additionally, GFAT1 activity and O-GlcNAc levels undergo changes following exposure to cisplatin; protein O-GlcNAcylation has no significant impact on the anti-tumor activity of cisplatin [54].

In lung cancer cells, inhibition of GFAT1 activity reduces PD-L1 glycosylation, accelerates its degradation, and diminishes PD-L1-induced suppression of NK and T cells [55]. Consequently, GFAT1 activity inhibition enhances immune cell activity against cancer cells. GFAT2 is also implicated in lung cancer cell proliferation, metastasis, and invasion. It is essential for metabolic reprogramming in non-small-cell lung cancer (NSCLC), especially in tumors harboring concurrent mutations in the tumor suppressor STK11 (LKB1) and the oncogene KRAS [56].

In summary, the role of GFAT in lung cancer underscores its therapeutic potential. By regulating the HBP, GFAT1 not only promotes tumor survival and progression under nutrient stress but also supports critical mechanisms such as EMT, chemoresistance, and immune evasion. GFAT1 inhibition presents a multifaceted strategy to suppress tumor growth by impairing glycosylation-dependent pathways essential for cancer cell survival, including PD-L1 stabilization and chemotherapy resistance mediated by the endoplasmic reticulum stress response. Moreover, the reliance of KRAS/LKB1 co-mutant tumors on GFAT2 for metabolic reprogramming reveals a distinct vulnerability that can be exploited for targeted therapy.

### GFAT in breast cancer

As a highly prevalent and clinically challenging malignancy worldwide, breast cancer constitutes a major public health burden [57], with substantial morbidity and mortality persisting despite significant advances in conventional therapies [58]. Both GFAT1 and GFAT2 have been reported to be overexpressed in breast cancer, with elevated GFAT expression correlating with unfavorable clinical outcomes [59], including increased tumor size, reduced progression-free and overall survival, and diminished pathologic complete response rates to neoadjuvant chemotherapy in triple-negative breast cancer (TNBC) patients [60].

In breast cancer, GFAT1 contributes to the formation of a tumor-promoting microenvironment [59] through its involvement in hyaluronan synthesis and can promote cancer stem cell (CSC)-like properties. Forced GFAT1 protein expression drives increased HA synthesis and activates hypoxia-inducible factor 1 (HIF-1) signaling, which not only accelerates the glycolytic program but also sustains the CSC-like subpopulation in breast cancer cells [61]. Another mechanism by which GFAT promotes cancer stem cell (CSC)-like properties via HA involves the Akt/GSK3β/β-catenin pathway. Additionally, its role in protein O-GlcNAcylation contributes to the modulation of CSC-like features through overlapping but distinct mechanisms from hyaluronan signaling [62].

Nicotine (Nic) is a key component of tobacco, implicated in the promotion of EMT in breast cancer cells. Increased O-GlcNAcylation of the transcriptional repressor C/EBP homologous protein (CHOP) through upregulation of GFAT expression inhibits its heterodimerization with CCAAT/enhancer-binding protein B (CEBPB), hence enhancing CEBPB’s DNA-binding activity and establishing a positive feedback loop that promotes EMT in response to Nic stimulation [63].

GFAT can also promote aggressive cancer behaviors in breast cancer by mediating O-GlcNAcylation of MORC family CW-type zinc finger 2 (MORC2) [64, 65]. Transforming growth factor-β1 (TGF-β1) enhances the stability of GFAT, which in turn increases MORC2 O-GlcNAcylation through the HBP. This modification of MORC2 facilitates the upregulation of key pro-tumorigenic genes such as snail family transcriptional repressor 1 (SNAIL) and connective tissue growth factor (CTGF), enhancing aggressive cancer behaviors [66].

In conclusion, GFAT contributes to hyaluronan synthesis, which fosters a pro-tumorigenic microenvironment and supports cancer stem cell-like phenotypes. Additionally, GFAT-mediated O-GlcNAcylation influences key signaling pathways, such as Akt/GSK3β/β-catenin axis, and modifies proteins like MORC2, facilitating aggressive cancer behaviors, including metastasis and invasion.

### GFAT in liver cancer

Liver cancer is among the most commonly diagnosed malignancies, with hepatocellular carcinoma (HCC) comprising the predominant subtype, representing approximately 70% to 90% of primary liver cancer cases [67]. Among HCC tumors, higher GFAT1 expression correlates with worse clinicopathological features and poorer patient survival. Functional studies have revealed that GFAT1 promotes HCC cell proliferation, migration, invasion, and cell cycle progression [68].

Knockout or pharmacologic inhibition of GFAT reduces cancer cell growth and increases the susceptibility of a subset of cancer cells to apoptosis under conditions of diamide-induced oxidative stress [69]. GFAT1 is also involved in liver cancer caused by increased O-GlcNAcylation levels in phosphoenolpyruvate carboxykinase 1 (PCK1)-loss hepatoma cells. Mechanistically, the loss of PCK1 activates the AMPK-GFAT1 axis, further promoting UDP-GlcNAc production and elevating O-GlcNAcylation levels. Importantly, reduced PCK1 expression enhances the O-GlcNAcylation of CHK2 at threonine 378, which in turn increases phosphorylation of the retinoblastoma protein (Rb) and promotes HCC cell growth [70]. HBV induces significant metabolic reprogramming in host cells, including the biosynthesis of hexosamine and phosphatidylcholine through the activation of *GFPT1* and choline kinase alpha (*CHKA*) at the transcriptional level respectively [71].

In short, GFAT1 not only drives HCC cell proliferation, migration, and invasion but also enhances the aggressive phenotypes associated with metabolic vulnerabilities such as PCK1 loss. Moreover, its involvement in HBV-associated HCC underscores its role in virus-driven metabolic alterations and oncogenesis, highlighting the dual importance of GFAT1 in tumor biology and viral replication.

### GFAT in melanoma

Malignant melanoma is a highly aggressive cancer originating from pigment-producing melanocytes [72]. Elevated protein levels of GFAT1 and subsequent increases in UDP-GlcNAc levels have been identified as being linked to enhanced hyaluronan production in both early-stage and advanced human melanomas, correlating with tumor progression. GFAT1 helps maintain a steady production of UDP-GlcNAc, which in turn influences hyaluronan synthesis through multiple mechanisms: serving as a direct precursor in hyaluronan biosynthesis, enhancing the O-GlcNAcylation of HAS3, modulating HAS3 lysosomal degradation, and prolonging HAS3 retention at the plasma membrane [73].

In melanoma cells, deletion of *GLO1*, which encodes glyoxalase 1—a detoxifying enzyme for the glycolytic byproduct methylglyoxal (MG)—results in downregulation of gene expression of *GFPT1* and *GFPT2*, compromised glucose metabolism, and depletion of metabolites required for glycosylation. This metabolic disruption correlates with altered oxidative stress responses and a shift in cellular dynamics, including enhanced tumor proliferation and metastasis in vivo [74].

Despite advancements in therapeutic strategies, a definitive treatment for advanced melanoma has yet to be established. Anandamide (AEA)-mediated treatment of metastatic melanoma cells results in a reduction in *GFPT1* expression, which subsequently reduces the production of glycoproteins such as L1-CAM. This alteration in the glycosylation profile results in lower cell migration, thereby diminishing the metastatic ability of melanoma cells [75]. Thus, targeting GFAT1-mediated pathways, particularly through the use of AEA, may represent a promising therapeutic strategy for limiting melanoma progression and metastasis.

### GFAT in other cancers

In gastric cancer tissues, GFAT1 expression is terribly reduced, with low levels correlating with more aggressive tumor features such as vessel invasion, advanced T stage, lymph node and distant metastasis, advanced TNM stage, and poor prognosis. Mechanistically, the downregulation of GFAT1 protein expression promotes EMT by inducing TGF-β1 expression, which enhances the invasive potential of gastric cancer cells [76].

In glioma patients, elevated GFAT1 levels are associated with higher malignancy grades and correlate with poor survival outcomes. Research has found that GFAT1 can promote the metastasis of U-118 cell [77]. MTOR2, rather than MTOR1, enhances GFAT1 activity, leading to enhanced glucosamine-6-phosphate synthesis independent of the PI3K/AKT pathway. High glucose or glutamine levels further promote MTOR2 activity. In turn, the upregulation of glycolytic and glutaminolytic metabolism disrupts MTOR dimerization, which facilitates the dissociation of MTOR2 from the MTOR complex. As a transcriptional factor, C-MYC, directly targeted by MTOR2, upregulates the mRNA expression of *GFPT1*. Collectively, the MTOR2/C-MYC/GFAT1 axis emerges as a pivotal mechanism in glioma progression [77].

In prostate cancer cells, inhibition of Myc unexpectedly leads to GFAT1 upregulation by the IREα-Xbp1s signaling pathway, resulting in enhanced protein glycosylation through the HBP. Myc inhibitor such as 10074 or 10058, together with a GFAT1 inhibitor such as 6-diazo-5-oxo-L-norleucine (DON), exert a synergistic antitumor effect in suppressing the proliferation and migration while inducing apoptosis of prostate cancer cells. Additionally, the delivery of a novel prodrug, 10074-DON, which simultaneously inhibits Myc and GFAT1, via a polysaccharide-based nanocarrier (PS), significantly improves tumor growth suppression and enhances the immune microenvironment in prostate cancer models, suggesting that concomitant inhibition of Myc and GFAT1 may exert synergistic antitumor effects [78].

In bladder cancer (BCa), GFAT1, secreted by tumor cells via small extracellular vesicles (sEVs), reprograms endothelial cell (EC) metabolism by enhancing the HBP flux, which results in elevated O-GlcNAcylation, especially in the nutrient-deprived tumor microenvironment. This modification affects the stability and nuclear translocation of seryl-tRNA synthetase (SerRS), a key regulator of angiogenesis. Specifically, O-GlcNAcylation at Ser101 promotes SerRS degradation by enhancing ubiquitination and hinders nuclear import via importin α5 recognition of the NLS motif, which allows vascular endothelial growth factor (VEGFA) transcription to proceed unopposed [79].

In colorectal cancer (CRC), the hyperactivity of the GFAT contributes to tumor malignancy. High glucose concentrations drive GFAT overproduction, leading to aberrant glycosylation that fuels cancer cell proliferation, metastasis, and invasion in colon cancer [80].

In cholangiocarcinoma (CCA), high glucose levels promote cancer progression by increasing the protein expressions of GFAT and vimentin, leading to enhanced O-GlcNAcylation of proteins, which correlates with increased migration and invasion of CCA cells, particularly in highly metastatic sublines [81].

In cervical cancer, GFAT1 promotes cell proliferation via regulating the ubiquitination and degradation of Phosphatase and tensin homolog (PTEN).

## GFAT in cardiovascular disease (CVD)

**Fig. 4:**
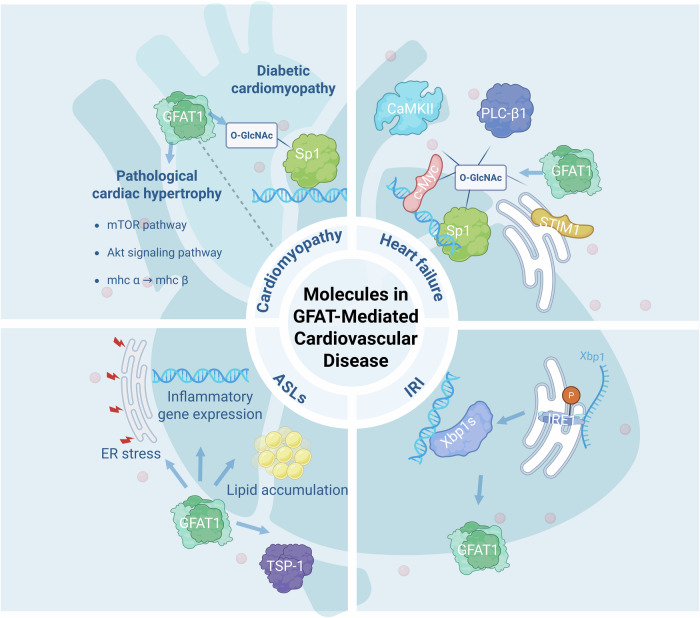
Molecules in GFAT-mediated cardiovascular disease. GFAT1 is a key driver of early hypertrophic growth through mTOR activation, while GFAT2 predominantly activates the Akt pathway, promoting pro-hypertrophic signaling. The temporal regulation of GFAT1 during the progression from early hypertrophy to heart failure highlights its stage-specific role. In diabetic cardiomyopathy, chronic hyperglycemia-induced GFAT activation leads to excessive O-GlcNAcylation, which drives pathological changes, including fibrosis, apoptosis, and inflammation, while limiting the cardioprotective effects of rIPC. In heart failure, GFAT-mediated O-GlcNAcylation of calcium-handling proteins contributes to arrhythmias, contractile dysfunction, and adverse remodeling. During ischemia/reperfusion injury, GFAT1, regulated by Xbp1s, promotes HBP flux and O-GlcNAcylation, which can exert a cardioprotective effect.

This chapter delves into the multifaceted role of GFAT in cardiovascular disease, exploring its contributions to cardiomyopathy, heart failure, ischemia/reperfusion injury, and atherosclerotic lesions (Fig. 4).

### GFAT in cardiomyopathy

This section explores the dual role of GFAT in pathological cardiac hypertrophy and diabetic cardiomyopathy, emphasizing its isoform-specific contributions and potential as a therapeutic target.

#### GFAT in pathological cardiac hypertrophy

Pathological cardiac hypertrophy initially acts as a compensatory mechanism to preserve heart function under mechanical or neurohormonal stress [82] by inducing ventricular thickening to reduce wall stress and temporarily support cardiac performance [70]. However, prolonged hypertrophy becomes maladaptive, eventually leading to heart failure [83, 84]. One key factor in this progression is metabolic derangement [85–89]. While the normal heart primarily relies on fatty acids for energy production, hypertrophied hearts shift toward increased glucose uptake, with a 50% rise in glucose consumption [90, 91]. Despite this, glucose oxidation through the tricarboxylic acid cycle remains unchanged [92], suggesting that excess glucose is redirected to alternative pathways like the HBP. Supporting this, elevated levels of UDP-GlcNAc have been observed in pressure-overloaded rat hearts [93], along with increased O-GlcNAc protein modification in various hypertrophy models [94]. The development of cardiac hypertrophy is governed by numerous intricate intracellular signaling pathways [84, 95], within which GFAT plays a crucial regulatory role.

GFAT1 is upregulated in cardiomyocytes during hypertrophic growth, and its overexpression promotes pathological cardiac remodeling, fibrosis, and dysfunction in response to pressure overload. Mechanistically, high protein levels of GFAT1 lead to increased O-GlcNAcylation and activation of the mTOR pathway, which exacerbates hypertrophy and drives the transition from compensatory growth to heart failure [96]. GFAT2 has been identified as the predominant isoform of GFAT in the heart. Elevated protein levels of GFAT2 facilitate isoproterenol (ISO)-induced cardiomyocyte hypertrophy by enhancing Akt O-GlcNAcylation and activation [97].

During cardiac hypertrophy, activation of AMP-activated protein kinase (AMPK) primarily inhibits the enzymatic activity of GFAT through phosphorylation, thereby reducing the O-GlcNAcylation of proteins, including troponin T, to suppress hypertrophy [98]. Moreover, GFAT can regulate mhc isoform switching during pressure overload-induced hypertrophy. Increased glucose uptake during pressure overload leads to elevated intracellular levels of UDP-N-acetylglucosamine, which promotes the expression of fetal genes such as myosin heavy chain beta (mhcβ), while suppressing the adult isoform mhcα. Interestingly, while *GFPT2* expression increases under these conditions, *GFPT1* does not change significantly, suggesting isoform-specific regulation. Moreover, restriction of dietary glucose can abolish this mhc isoform switching, which highlights the regulatory role of glucose metabolites in isoform switching of sarcomeric proteins characteristic for the fetal gene program [93].

Pressure-overload hypertrophy (POH) is a type of pathological cardiac hypertrophy. GFAT1-induced O-GlcNAcylation is essential for early hypertrophic growth but not for the continuation of hypertrophy during prolonged pressure overload. GFAT1 protein levels rise significantly during the early phase of hypertrophic growth (1 week after transverse aortic constriction, TAC), leading to increased protein O-GlcNAcylation, which promotes active myocardial hypertrophy and remodeling. However, as hypertrophy stabilizes (6 weeks after TAC), protein O-GlcNAc levels decline, and augmenting them during this later stage does not restart hypertrophic growth. These findings highlight the temporal regulation of GFAT1 [99].

In conclusion, GFAT1 is pivotal in the early phase of hypertrophic growth, promoting myocardial remodeling and mTOR activation, whereas GFAT2 primarily regulates pro-hypertrophic signaling through the Akt pathway. Temporal and isoform-specific regulation of GFAT emphasizes its role in metabolic reprogramming during hypertrophy, including fetal gene reactivation and mhc isoform switching.

#### GFAT in diabetic cardiomyopathy (DC)

DC initially manifests as diastolic left ventricular (LV) dysfunction and later progresses to systolic dysfunction with cardiac hypertrophy and fibrosis [100], ultimately culminating in congestive heart failure [101]. Diabetes-induced hyperglycemia drives the upregulation of *GFPT1* and *GFPT2* gene expression [102], correlating with cardiomyocyte hypertrophy, fibrosis, apoptosis, and inflammation [103]. Mechanistically, increased GFAT levels correlate with elevated O-GlcNAcylation of cardiac proteins and transcription factor Sp1 [104], contributing to various metabolic effects of sustained high glucose levels, such as reduced expression of sarcoplasmic reticulum Ca(2 + )-ATPase [105] in cardiomyocytes and upregulation of TGF-β and plasminogen activator inhibitor-1 in vascular smooth muscle cells, mesangial cells, and endothelial cells [106]. Fatty acids also contribute to GFAT upregulation, further driving excessive O-GlcNAcylation [102].

A study has examined the role of GFAT in O-GlcNAcylation and its impact on cardioprotection through remote ischemic preconditioning (rIPC) in non-diabetic and diabetic hearts. rIPC increases O-GlcNAc levels and improves post-ischemic recovery in non-diabetic hearts. However, in type 2 diabetes, where O-GlcNAc levels are already elevated, the additional protection from rIPC is limited. Importantly, inhibition of GFAT with azaserine abolishes both the increase in O-GlcNAc levels and the cardioprotective effects, indicating that GFAT-mediated O-GlcNAcylation is essential for rIPC-induced cardioprotection and contributes to the chronic activation of protective signaling in diabetic myocardium [107].

Collectively, GFAT plays a central role in the pathogenesis of DC by driving excessive O-GlcNAcylation, which disrupts key cellular processes in the heart. While cardioprotective strategies like rIPC leverage O-GlcNAcylation, chronic GFAT-mediated activation of this pathway in diabetes limits their efficacy.

### GFAT in heart failure

People with diabetes between the ages of 45 and 65 face a significantly increased risk of cardiovascular death [108]. O-GlcNAcylation of calmodulin-dependent protein kinase II (CAMKII) in cardiomyocytes, particularly at S279 [109], is linked to the arrhythmogenic effects of diabetes, including decreased contractility and higher rates of cardiac arrhythmias. O-GlcNAcylation of proteins like troponin T (TnT) [110] has been shown to be linked to reduced phosphorylation and impaired calcium sensitivity in the heart, which may contribute to cardiac dysfunction in hypertrophy and heart failure. Furthermore, other Ca^2+^ signaling pathways, such as PLC-β1 [111], STIM1 [112], and hypertrophic signaling induced by angiotensin II [113], are also regulated by O-GlcNAcylation in an HBP-dependent manner [114].

O-GlcNAcylation has complex effects, exerting both pro- and anti-hypertrophic influences by involving transcription factors like Sp1 [115] and c-Myc and signaling molecules such as Ca^2+^ and STIM1 [116]. Moreover, microRNA-539 has been identified as a regulator of OGA levels [117], potentially linking O-GlcNAcylation to epigenetic regulation in heart disease. However, the role of GFAT in these processes remains to be investigated.

### GFAT in ischemia/reperfusion injury (IRI)

Ischemia and reperfusion injury (IRI) underlies the pathophysiology of various conditions, including cerebral ischemia, myocardial infarction, hemorrhagic shock, stroke, and organ transplantation (among others) [118–124]. GFAT1 is required for the spliced X-box binding protein 1 (Xbp1s)-mediated cardioprotective response. In response to ischemia/reperfusion (I/R) injury, the unfolded protein response (UPR), particularly through Xbp1s, plays a key protective role by upregulating *GFPT1* transcription, enhancing the HBP, and increasing protein O-GlcNAcylation [125]. Glutamine pre-treatment can significantly improve heart function during reperfusion and reduce cardiac injury, as associated with increased protein O-GlcNAc and ATP levels [126].

### GFAT in atherosclerotic lesions

GFAT exacerbates ER stress under hyperglycemia, driving lipid accumulation and inflammation, which are linked to the pathogenesis of hepatic steatosis and atherosclerosis in diabetic conditions. In human aortic smooth muscle cells (HASMC) elevated glucose upregulates thrombospondin-1 (TSP-1) transcription through protein glycosylation driven by GFAT activation to promote atherosclerosis [127]. Moreover, in HepG2 hepatic cells with overexpressing GFAT, increased HBP flux has been found to trigger the unfolded protein response (UPR) [128].

## GFAT in Diabetes Mellitus (DM)

**Fig. 5:**
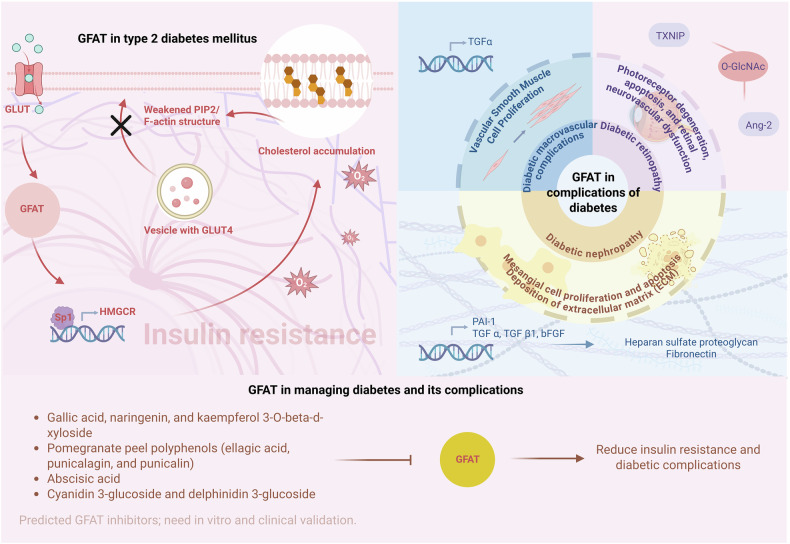
Roles of GFAT in type 2 diabetes mellitus and its complications. Elevated GFAT activity in response to chronic hyperglycemia leads to elevated production of UDP-GlcNAc, resulting in excessive O-GlcNAcylation of key insulin signaling proteins, impaired GLUT4 translocation, and insulin resistance. Moreover, GFAT-mediated HBP activation disrupts plasma membrane integrity via cholesterol accumulation and cytoskeletal remodeling, further compromising glucose transport. Beyond insulin resistance, GFAT is implicated in diabetic complications such as retinopathy and macrovascular disease, where it promotes endothelial dysfunction, inflammation, and vascular remodeling through increased protein O-GlcNAcylation. In diabetic retinopathy, GFAT2 upregulation drives photoreceptor degeneration and neovascularization, whereas in vascular smooth muscle cells, GFAT enhances TGFα transcription, accelerating atherosclerosis.

In type 2 diabetes mellitus (T2DM), elevated GFAT activity exacerbates metabolic dysfunctions, including insulin resistance and impaired glucose handling. These disruptions are further compounded by GFAT’s involvement in nutrient sensing and oxidative stress regulation. Additionally, GFAT contributes significantly to the pathological mechanisms underlying diabetes-related complications, such as macrovascular damage, retinopathy, and nephropathy (Fig. 5).

This chapter delves into GFAT’s central role in DM, exploring its contribution to T2DM and the complications arising from persistent hyperglycemia. Additionally, this chapter examines the therapeutic potential of targeting GFAT to mitigate these effects, which emphasizes recent advances in modulating GFAT activity through natural compounds to improve metabolic outcomes and reduce diabetes-related tissue damage.

### GFAT in T2DM

Insulin resistance, a hallmark and predictor of T2DM [129], is characterized by the inability of insulin to effectively stimulate glucose transport and glycogen synthesis, resulting in elevated blood glucose levels and subsequent metabolic dysfunction. High levels of glucose have been found to drive increased GFAT activity, resulting in excessive production of UDP-GlcNAc, which can disrupt normal insulin signaling pathways and promote insulin resistance [130]. Overexpression of GFAT can also induce insulin resistance [131]. Reducing GFAT activity by inhibitors can completely block glucose-induced desensitization of the insulin-responsive GTS. However, these inhibitors do not prevent glucosamine-induced desensitization [132].

Glucose transport serves as the rate-limiting step in skeletal muscle glucose metabolism. This process occurs through facilitated diffusion, relying on two distinct glucose transporter isoforms: GLUT1 and GLUT4. Increased glucose transport via the GLUT1 pathway leads to enhanced GFAT activity, not its mRNA abundance, contributing to glycogen accumulation and insulin resistance [133]. Increased glucose flux through heightened GFAT activity can also disrupt plasma membrane PIP2-regulated F-actin structures via cholesterol accumulation, impairing glucose transporter-4 function and contributing to insulin resistance. The translocation of GLUT4 from intracellular vesicles to the plasma membrane is a complex process involving lipid signaling (PIP₂) and cytoskeletal remodeling (F-actin). Increased HBP activity has been further identified as leading to cholesterol accumulation in the PM, which in turn weakens the PIP2/F-actin structure [134]. In vivo, a Western-style high-fat diet leads to a rapid accumulation of cholesterol in skeletal muscle plasma membranes and subsequent insulin resistance within just one week, facilitated by O-GlcNAcylation via increased transcriptional activity of the Sp1, which enhances the expression of hydroxy-3-methylglutaryl CoA reductase (HMGCR). The resulting elevation in plasma membrane cholesterol is associated with a loss of cortical F-actin. Thus, targeting the HBP and its downstream effects on cholesterol metabolism could offer new therapeutic strategies for preventing or treating type 2 diabetes [135].

Hexosamine pathway activation, however, has been found to contribute to beta-cell dysfunction observed in diabetes through oxidative stress rather than O-linked glycosylation [136]. Overexpression of GFAT or treatment with glucosamine can lead to impaired glucose-stimulated insulin secretion and decreased expression of key beta-cell genes, including insulin, GLUT2, and glucokinase. The transcription factor PDX-1, essential for regulating these genes, also exhibits reduced DNA binding activity [136].

GFAT is essential for glucose-induced upregulation of tribbles homolog 3 (TRIB3), a central regulator of glucose-induced insulin resistance. Furthermore, glucosamine, which bypasses GFAT and directly enters the HBP, can also increase TRIB3 expression and reduce insulin sensitivity [137].

A study explored whether the HBP can act as a nutrient-sensing mechanism. Elevated levels of UDP-hexosamines (UDP-GlcNAc and UDP-GalNAc) were correlated with reduced insulin-stimulated glucose uptake. However, cellular UDP-hexosamines do not significantly increase with higher glucose intake. Despite insulin-stimulated glucose uptake being impaired in glucosamine-treated cells, prolonged glucose exposure in the presence of insulin only marginally increased UDP-hexosamine levels across all glucose concentrations [138]. Therefore, while UDP-hexosamines contribute to glucosamine-induced insulin resistance, they do not act as nutrient sensors in glucose-treated adipocytes, raising doubts about the role of HBP in glucose-induced insulin resistance.

### GFAT in complications of diabetes

The complications of diabetes are multifaceted and affect various organ systems, with vascular, retinal, and renal pathologies being among the most debilitating. This section explores the critical involvement of GFAT in the development of diabetic macrovascular complications, retinopathy, and nephropathy, detailing the underlying mechanisms and clinical implications of its activity in these conditions.

#### GFAT in Diabetic Macrovascular Complications

Strong epidemiologic evidence has linked hyperglycemia to vascular disease in type I diabetes [139, 140], with early-stage atherosclerosis characterized by enhanced proliferation of vascular smooth muscle cells. Growth factors like TGFα and basic fibroblast growth factor have been hypothesized to play critical roles in this process [141–144]. GFAT is crucial to the regulation of TGFα gene transcription in response to glucose, not just by fueling glycosylation processes but also by potentially modulating transcription factor function through O-GlcNAc modifications [145].

#### GFAT in diabetic retinopathy (DR)

The enzyme GFAT, particularly its GFAT2 isoform, has been shown to be upregulated in diabetic retinas and in cells exposed to high-glucose conditions, promoting increased O-GlcNAc modification. This modification is associated with photoreceptor degeneration, apoptosis, and retinal neurovascular dysfunction—key features of DR. Activation of AMPK, a central regulator of energy metabolism, can downregulate the GFAT/TXNIP-O-GlcNAc modification signaling axis, thereby reducing photoreceptor degeneration and inhibiting neovascularization in DR [146]. High-fat diet (HFD) consumption can elevate retinal O-GlcNAcylation by facilitating GFAT2 expression and increased HBP flux [147]. GFAT2 upregulation is also linked to increased expression of the nuclear receptor subfamily 4 group A member 1 (NR4A1, alternatively referred to as NGF1B, TR3, and Nur77). Importantly, increased O-GlcNAcylation in the retina may precede the development of diabetes, indicating that retinal changes can occur before the onset of hyperglycemia or other diabetic symptoms.

GFAT is linked to retinal endothelial damage in diabetic retinopathy (DR). In endothelial cells (ECs), hyperglycemia or nucleoside diphosphate kinase-B (NDPK-B) deficiency leads to an increase in GFAT expression and phosphorylation, which is associated with the upregulation of O-GlcNAcylated angiopoietin-2 (Ang-2), a factor inducing pericyte loss, retinal vasoregression, and acellular capillary formation [148]. Consequently, GFAT, particularly GFAT2, has been emerging as a potential therapeutic target for interventions aimed at reducing O-GlcNAcylation and delaying the progression of diabetic retinopathy.

#### GFAT in Diabetic Nephropathy (DN)

As a prominent complication of diabetes, diabetic nephropathy (DN) represents a leading cause of end-stage renal disease [149]. Increased GFAT expression has been observed in diabetic glomeruli [150], particularly in mesangial cells (MCs) [151], which leads to the overproduction of cytokines such as transforming growth factor-β (TGF-β1), TGF-α, and basic fibroblast growth factor (bFGF). These cytokines are key mediators of renal extracellular matrix synthesis [150]. Specifically, high glucose concentrations can activate glucose-response elements (GlREs) in the TGF-β1 promoter, with the upstream stimulatory factors (USF-1 and USF-2) binding to these elements, increasing TGF-β1 transcription. GFAT overexpression further amplifies this effect by increasing USF-2 expression and its binding to the TGF-β1 promoter [152]. The activation of TGF-β1 by GFAT’s regulation of glucose metabolism is partly mediated through protein kinase C (PKC) signaling. Moreover, GFAT activity is tightly controlled by cyclic adenosine monophosphate (cAMP)-dependent phosphorylation, which points to a complex regulation of GFAT through phosphorylation [150]. Glucosamine, a downstream metabolite of GFAT, is even more potent than glucose in promoting TGF-β1 expression and its activation [153]. GFAT upregulation directly stimulates TGF-β1 expression at the transcriptional level. Notably, TGF-β2 production is unaffected, highlighting the specificity of GFAT’s regulatory effects on TGF-β1 [154].

GFAT overexpression in MC also significantly increases the activity of the plasminogen activator inhibitor-1 (PAI-1) promoter, especially under high glucose concentrations, leading to increased PAI-1 gene expression. This increase in PAI-1 contributes to vascular injury and extracellular matrix accumulation, hallmark features of diabetic glomerulosclerosis. GFAT overexpression also amplifies the activation of vascular injury pathways independent of TGF-β1 [155].

Angiotensin II (ANG II), a key player in diabetic kidney disease, has been demonstrated to upregulate GFAT promoter activity, mRNA, and protein levels via the ANG II type I receptor (AT1). The process is calcium-dependent and involves various signaling pathways, including protein kinase C (PKC), protein tyrosine kinase (PTK), Src family kinases, the EGFR, and p42/44 MAPK. By enhancing GFAT expression, ANG II amplifies glucose flux through the hexosamine pathway, which contributes to the expression of genes associated with vascular and glomerular injury [151].

GFAT plays an essential role in driving mesangial cell proliferation and apoptosis in response to hyperglycemia through mTOR signaling, contributing to the pathophysiology of diabetic nephropathy [156].

### GFAT in managing diabetes and its complications

GFAT was studied earliest in diabetes [157] and is targeted by the widest range of natural compounds in this field, providing a strong foundation for developing safe and effective interventions, unlike in cancer, cardiovascular disease, or neurodegenerative disorders. This section delves into the role of GFAT in diabetes management, highlighting innovative therapeutic strategies and the potential of natural compounds to alleviate metabolic dysregulation and its complications, while also noting the need for further in vitro and clinical studies to confirm their therapeutic efficacy.

The antidiabetic properties of *Anacardium occidentale* have been well established [158]. Phytochemicals from *Anacardium occidentale*, including gallic acid, naringenin, and kaempferol 3-O-beta-d-xyloside, were predicted to be potential inhibitors of two key therapeutic targets, GFAT1 and DPP-4, in the management of diabetes, based on molecular docking, binding free energy, QSAR and ADME analyses [159], which, however, are insufficient to confirm their therapeutic efficacy against GFAT1 and DPP-4, and further experimental validation is required to substantiate their inhibitory potential.

An in silico analysis was conducted to evaluate three *Punica granatum* peel metabolites, punicalin, punicalagin, and ellagic acid, which exhibited significant binding affinities with multiple protein targets, including GFAT, PTP1β, PPAR-ᵞ, TKIR, RBP4, α-amylase, α-glucosidase, GCK, and AQP-2 [160].

Abscisic acid (ABA), a bioactive compound found in okra, has been evaluated for its potential to inhibit GFAT through in silico molecular docking. ABA exhibited a low binding energy (∆G = −7.3 kcal/mol) with GFAT, indicating strong binding affinity and potential inhibitory action. The favorable pharmacokinetic and ADMET properties of ABA suggest that it has the potential to be an effective, natural anti-diabetic agent with minimal toxicity [161].

Polyphenols, particularly anthocyanins found in blue corn and black bean extracts, have been identified as interacting with several key proteins involved in type 2 diabetes mellitus (T2DM), including GFAT. In silico molecular docking has highlighted strong interactions of cyanidin 3-glucoside and delphinidin 3-glucoside with GFAT, as well as other proteins like 11β-HSD, PPARG, PTP, and RTKs. These proteins play roles in regulating inflammation, oxidative stress, insulin resistance, and glucose and lipid metabolism [162].

Vitamin D treatment has been shown to significantly improve hyperglycemia and insulin levels, while reducing the expression of AGER (AGE receptor) and OGT (O-linked N-acetylglucosamine transferase), both of which are associated with diabetic complications. Within the HBP, both *OGT* and *GFPT* gene expression levels were elevated in the diabetic group; however, only *OGT* expression was restored following vitamin D treatment [163, 164].

In diabetic nephropathy (DN), rhein, an anthraquinone compound derived from rhubarb, has been shown to effectively treat experimental DN [165]. Rhein reduced high glucose-induced GFAT activity in rat mesangial cells but did not directly inhibit the GFAT enzyme itself. By inhibiting activation of the HBP, rhein downregulated TGF-β1 and p21 expression, thus attenuating cellular hypertrophy and extracellular matrix synthesis [166].

In conclusion, although these natural compounds have predicted in silico to interact with GFAT, their actual inhibitory effects on GFAT remain unverified. Systematic mechanistic studies in appropriate cell culture models are required to confirm whether these compounds directly modulate GFAT activity or affect diabetes through alternative mechanisms.

## GFAT in degenerative diseases

This section examines the role of GFAT in neurodegenerative diseases such as Alzheimer’s. Additionally, it explores the impact of GFAT on osteoarthritis (OA) and post-traumatic osteoarthritis (PTOA), highlighting its role in regulating cartilage homeostasis and inflammation.

### GFAT in neurodegenerative diseases

GFAT1 plays a critical role in the link between altered brain metabolism and Alzheimer’s disease (AD) progression, particularly under a high-fat diet (HFD). HFD-induced insulin resistance triggers AMPK-driven suppression of GFAT1 enzyme activity, which leads to impaired flux through the HBP [167]. Global protein O-GlcNAcylation is markedly diminished in the Alzheimer’s cerebrum [168]. Notably, reduced O-GlcNAc modification, specifically of tau protein and mitochondrial Complex I subunit NDUFB8 [167], correlates negatively with tau hyperphosphorylation [168], thereby promoting tau toxicity, mitochondrial dysfunction, and the accumulation of neurofibrillary tangles, key hallmarks of AD [169, 170].

### GFAT in osteoarthritis (OA)

GFAT1 protein expression in osteoarthritis is significantly decreased [171]. GFAT plays a key role in countering the effects of IL-1β-induced cartilage degeneration, a hallmark of osteoarthritis (OA). When synoviocytes overexpressing GFAT were co-cultured with chondrocytes and challenged with IL-1β, the typical IL-1β-induced decrease in proteoglycan synthesis and increase in NO and PGE2 levels were prevented. This protective effect is attributed to increased production of glucosamine-6-phosphate, the metabolite into which exogenous glucosamine is first converted upon entering the cell. Initial findings indicate that certain slow-acting drugs, such as glucosamine, may positively impact the progression of OA [172, 173]. Overexpression of GFAT could replicate the protective effects of glucosamine without requiring high exogenous glucosamine doses, which offers a novel approach for OA treatment by modulating the hexosamine pathway within joint tissues [174].

Joint degeneration is strongly influenced by prior injuries, which serve as a significant determinant in the onset of post-traumatic OA (PTOA), a condition that can develop in younger individuals and accounts for approximately 10% of knee OA cases [175]. Cartilage trauma markedly alters HBP enzyme expression, reducing OGT and increasing OGA levels, thereby elevating the OGA/OGT ratio, while *GFPT1* mRNA levels are significantly decreased in highly degenerated cartilage. Treatments with GlcNAc and the OGA inhibitor PUGNAc improve chondrocyte viability after trauma, whereas the GFAT inhibitor AZA and high-dose glucosamine sulfate (GS) are cytotoxic [176].

## GFAT in other diseases

This chapter delves into the diverse roles of GFAT in other pathological conditions, including subarachnoid hemorrhage (SAH), hepatic encephalopathy (HE), chronic kidney disease (CKD), pulmonary arterial hypertension (PAH), coronavirus disease 2019 (COVID-19), methylmercury toxicity, acute myeloid leukemia (AML), and REM sleep deprivation (REMSD). By uncovering the multifaceted roles of GFAT in these diseases, we aim to provide a comprehensive understanding of its significance in human health and pathology, paving the way for novel therapeutic strategies.

### GFAT in hypercholesterolemia

In diet-induced hypercholesterolemia, global O-GlcNAcylation of liver proteins is decreased, accompanied by reduced expression of both OGT and GFAT1 [177]. GFAT1 expression is likely to be negatively regulated by certain metabolic disorders. For instance, glutamine withdrawal can induce decreased GFAT1 expression and reduced HBP output through an mTORC2-XBP1s axis [178].

### GFAT in subarachnoid hemorrhage (SAH)

SAH can induce severe neuroinflammation and ER stress, resulting in initiation of the UPR. The IRE1/XBP1s signaling pathway plays a central role in this response, with Xbp1s upregulating *GFPT1* transcription. Elevated GFAT1 protein expression, in turn, increases O-GlcNAc levels, which have a protective effect on neural cells by enhancing their stress resilience [179].

### GFAT in hepatic encephalopathy (HE)

In HE, GFAT1 and GFAT2 are essential in driving oxidative stress and astrocyte senescence. Hyperammonemia, a hallmark of HE, upregulates GFAT1/2 protein expression, GlcN-6-P synthesis and O-GlcNAcylation-dependent inhibition of pri-miR326-3p transcription via a glutamine synthetase (GS)-dependent mechanism. Subsequently, glucosamine synthesis-dependent protein O-GlcNAcylation contributes to the pathogenesis of HE that triggers oxygen species (ROS), RNA oxidation and ER stress, as well as astrocyte senescence, through upregulation of HO1 and Nox4 [180].

### GFAT in Chronic Kidney Disease (CKD)

Reducing proteinuria has been proposed as a promising therapeutic strategy for CKD. Hypertensive conditions increase the expression of GFAT and O-GlcNAc transferase (OGT) in the renal cortex, which correlates with elevated O-GlcNAcylation and proteinuria. Specifically, this elevated O-GlcNAcylation impairs protein reabsorption in proximal tubule (PT) cells by reducing the surface expression of the key endocytic receptor megalin, which is crucial for albumin uptake [181].

### GFAT in pulmonary arterial hypertension (PAH)

In PAH, right ventricular dysfunction (RVD) is a key factor in disease progression and mortality [182–184]. Excess O-GlcNAcylation, linked to impaired mitochondrial function, is observed in the right ventricle (RV) of PAH patients and experimental models. Increased glucose uptake in PAH drives this pathway, worsening metabolic dysfunction in the RV. AMPK, which inhibits GFAT enzymatic activity by inducing its phosphorylation, reduces HBP intermediates and O-GlcNAcylation, restoring mitochondrial function and improving RV performance in monocrotaline (MCT) rats, but not in pulmonary artery-banded (PAB) rats. Thus, excess O-GlcNAcylation exacerbates metabolic abnormalities in the RV, and targeting GFAT through AMPK activation could be a therapeutic strategy in PAH [185].

### GFAT in coronavirus disease 2019 (COVID-19)

The potential role of glutamine metabolic reprogramming in SARS-CoV-2 infection and pathogenesis has been highlighted. SARS-CoV-2 may rely on enhanced anabolic metabolism, including altered glucose and glutamine utilization, similar to other viral infections and cancer cells, to meet its biosynthetic and energy demands. Key cellular factors such as GFAT1/2, GLS1, PSAT1, HIF-1α, mTORC1, and the transcription factor Myc are hypothesized to play a central role in glutamine metabolism during viral infection [186]. This metabolic shift could promote viral replication and dampen host immune responses. Although the exact mechanisms in SARS-CoV-2 remain unconfirmed, understanding these pathways could aid in developing targeted therapeutic strategies to disrupt viral proliferation.

### GFAT in methylmercury (MeHg) toxicity

GFAT has been identified as a key target molecule of methylmercury toxicity in yeast cells. GFAT catalyzes the production of glucosamine-6-phosphate, essential for the synthesis of amino sugars and glycoproteins, which are crucial for yeast cell survival. Methylmercury inhibits GFAT enzymatic activity by binding to its sulfhydryl (SH) group, leading to a loss of function. Yeast cells overexpressing GFAT exhibit resistance to methylmercury, suggesting that GFAT’s interaction with methylmercury mitigates its toxicity by reducing the availability of free methylmercury. The addition of glucosamine, which is converted into glucosamine-6-phosphate, also decreases methylmercury toxicity, further supporting GFAT’s role in mediating resistance [187, 188].

### GFAT in acute myeloid leukemia (AML)

AML cells have been reported to express elevated levels of HBP enzymes, including GFAT, and to exhibit increased O-GlcNAcylation, which is significant for maintaining their survival and undifferentiated state. In vivo experiments using xenograft AML models further demonstrate that inhibiting the HBP pathway significantly reduces tumor growth and clears tumor cells from bone marrow, spleen, and blood without causing systemic toxicity [189]. These findings suggest that targeting GFAT and the broader HBP pathway offers a promising therapeutic strategy for AML, addressing both the proliferation and differentiation block that characterizes this disease.

### GFAT in REM sleep deprivation (REMSD)

GAFT-mediated HBP flux and O-GlcNAcylation are critical for learning and memory (L/M) function and are markedly impaired by REM sleep deprivation (REMSD). REMSD reduces O-GlcNAc and OGT levels while increasing OGA expression and activity, particularly in the hippocampal dentate gyrus (DG), CA1, and cortex. These molecular changes are associated with decreased activation of the PKA/CREB, ERK, and C/EBP-β /C/EBP-δ signaling pathways following fear conditioning, leading to cognitive deficits. Inhibition of HBP flux with the GFAT inhibitor DON or suppression of OGT activity with OSMI-1 mimics REMSD effects, reducing O-GlcNAcylation and impairing L/M function [190].

## Outlook and Conclusion

GFAT is involved in diverse diseases, including cancer, cardiomyopathies, diabetes and its complications, neurodegeneration, kidney and lung disorders, and infectious diseases. It can regulate such cancer-related processes as proliferation, migration, invasion, apoptosis, stemness, and immune evasion, while also contributing to cardiac hypertrophy, fibrosis, arrhythmias, impaired insulin signaling and vascular function in diabetes, protein aggregation in neurodegeneration, and stress or detoxification responses. Mechanistically, GFAT can promote disease progression by increasing UDP-GlcNAc production, leading to dysregulated O-GlcNAcylation and N-glycosylation. GFAT is also linked to signaling pathways, including PI3K/AKT, ERK/MAPK, Wnt/β-catenin, and mTOR, and can disrupt DNA repair and calcium handling while promoting oxidative stress, fibrosis, and inflammation, though it may also confer protective effects like stress resistance and cardioprotection in specific contexts.

Contrary to the general notion that GFAT is upregulated under pathological or dysregulated conditions, reduced GFAT expression has been observed in several disease contexts, including gastric cancer, Alzheimer’s disease, osteoarthritis, hypercholesterolemia, and other metabolic disturbances.

Collectively, these findings underscore GFAT’s multifaceted role in human health and disease. However, its distinct functions in these diseases remain insufficiently defined.

In cancer, further research is needed to clarify the isoform-specific roles of GFAT1 and GFAT2, their influence on cancer stem cell (CSC) properties such as self-renewal and therapy resistance, and their crosstalk with other post-translational modifications (PTMs) like phosphorylation, lactylation, acetylation, and ubiquitination, which may significantly impact tumor behavior. Additionally, GFAT’s interaction with other metabolic pathways (e.g., the pentose phosphate pathway, lipid, and amino acid metabolism) remains underexplored and could offer new targets for disrupting cancer metabolism. In CVD, understanding the isoform-specific contributions of GFAT1 and GFAT2 in conditions like hypertrophy, heart failure, and diabetic cardiomyopathy is also essential, as their differential expression patterns and temporal dynamics during disease progression may offer time-specific therapeutic windows. The interplay between GFAT-driven O-GlcNAcylation and PTMs could also fine-tune cardiac signaling, making it a potential therapeutic avenue. In diabetes, GFAT’s role in beta-cell dysfunction and systemic metabolic dysregulation needs further exploration. Across these diseases, developing selective GFAT modulators and evaluating their efficacy in preclinical and clinical settings could lead to more precise, isoform- and tissue-specific therapies, ultimately improving disease management.

## Data Availability

No new data were created.

## References

[1] McKnight GL, Mudri SL, Mathewes SL, Traxinger RR, Marshall S, Sheppard PO, et al. Molecular cloning, cDNA sequence, and bacterial expression of human glutamine:fructose-6-phosphate amidotransferase. J Biol Chem. 1992;267:25208–12. 1460020

[2] Marshall S, Bacote V, Traxinger RR. Discovery of a metabolic pathway mediating glucose-induced desensitization of the glucose transport system. Role of hexosamine biosynthesis in the induction of insulin resistance. J Biol Chem. 1991;266:4706–12. 2002019

[3] Magalhães A, Duarte HO, Reis CA. The role of O-glycosylation in human disease. Mol Aspects Med. 2021;79:100964. 33775405 10.1016/j.mam.2021.100964

[4] Akella NM, Ciraku L, Reginato MJ. Fueling the fire: emerging role of the hexosamine biosynthetic pathway in cancer. BMC Biology. 2019;17:52. 31272438 10.1186/s12915-019-0671-3 PMC6610925

[5] Reily C, Stewart TJ, Renfrow MB, Novak J. Glycosylation in health and disease. Nat Rev Nephrol. 2019;15:346–66. 30858582 10.1038/s41581-019-0129-4 PMC6590709

[6] Chaveroux C, Sarcinelli C, Barbet V, Belfeki S, Barthelaix A, Ferraro-Peyret C, et al. Nutrient shortage triggers the hexosamine biosynthetic pathway via the GCN2-ATF4 signalling pathway. Sci Rep. 2016;6:27278. 27255611 10.1038/srep27278 PMC4891703

[7] Hart GW. Nutrient regulation of signaling and transcription. J Biol Chem. 2019;294:2211–31. 30626734 10.1074/jbc.AW119.003226 PMC6378989

[8] Hanover JA, Krause MW, Love DC. Bittersweet memories: linking metabolism to epigenetics through O-GlcNAcylation. Nat Rev Mol Cell Biol. 2012;13:312–21. 22522719 10.1038/nrm3334

[9] Vaidyanathan K, Durning S, Wells L. Functional O-GlcNAc modifications: implications in molecular regulation and pathophysiology. Crit Rev Biochem Mol Biol. 2014;49:140–63. 24524620 10.3109/10409238.2014.884535 PMC4912837

[10] Paneque A, Fortus H, Zheng J, Werlen G, Jacinto E. The Hexosamine Biosynthesis Pathway: Regulation and Function. Genes (Basel). 2023;14:933. 10.3390/genes14040933 PMC10138107 37107691

[11] Denzel MS, Storm NJ, Gutschmidt A, Baddi R, Hinze Y, Jarosch E, et al. Hexosamine pathway metabolites enhance protein quality control and prolong life. Cell. 2014;156:1167–78. 24630720 10.1016/j.cell.2014.01.061

[12] Horn M, Denzel SI, Srinivasan B, Allmeroth K, Schiffer I, Karthikaisamy V, et al. Hexosamine Pathway Activation Improves Protein Homeostasis through the Integrated Stress Response. iScience. 2020;23:100887. 32086012 10.1016/j.isci.2020.100887 PMC7033349

[13] Ruegenberg S, Horn M, Pichlo C, Allmeroth K, Baumann U, Denzel MS. Loss of GFAT-1 feedback regulation activates the hexosamine pathway that modulates protein homeostasis. Nat Commun. 2020;11:687. 32019926 10.1038/s41467-020-14524-5 PMC7000685

[14] Lam C, Low J-Y, Tran PT, Wang H. The hexosamine biosynthetic pathway and cancer: Current knowledge and future therapeutic strategies. Cancer Lett. 2021;503:11–8. 33484754 10.1016/j.canlet.2021.01.010

[15] Liu X, Blaženović I, Contreras AJ, Pham TM, Tabuloc CA, Li YH, et al. Hexosamine biosynthetic pathway and O-GlcNAc-processing enzymes regulate daily rhythms in protein O-GlcNAcylation. Nat Commun. 2021;12:4173. 34234137 10.1038/s41467-021-24301-7 PMC8263742

[16] Ghosh S, Blumenthal HJ, Davidson E, Roseman S. Glucosamine metabolism. V. Enzymatic synthesis of glucosamine 6-phosphate. J Biol Chem. 1960;235:1265–73. 13827775

[17] Milewski S. Glucosamine-6-phosphate synthase–the multi-facets enzyme. Biochim Biophys Acta. 2002;1597:173–92. 12044898 10.1016/s0167-4838(02)00318-7

[18] Teplyakov A, Obmolova G, Badet-Denisot MA, Badet B, Polikarpov I. Involvement of the C terminus in intramolecular nitrogen channeling in glucosamine 6-phosphate synthase: evidence from a 1.6 A crystal structure of the isomerase domain. Structure. 1998;6:1047–55. 9739095 10.1016/s0969-2126(98)00105-1

[19] Mouilleron S, Badet-Denisot M-A, Golinelli-Pimpaneau B. Glutamine binding opens the ammonia channel and activates glucosamine-6P synthase. J Biol Chem. 2006;281:4404–12. 16339762 10.1074/jbc.M511689200

[20] Floquet N, Mouilleron S, Daher R, Maigret B, Badet B, Badet-Denisot M-A. Ammonia channeling in bacterial glucosamine-6-phosphate synthase (Glms): molecular dynamics simulations and kinetic studies of protein mutants. FEBS Lett. 2007;581:2981–7. 17559838 10.1016/j.febslet.2007.05.068

[21] Raczynska J, Olchowy J, Konariev PV, Svergun DI, Milewski S, Rypniewski W. The crystal and solution studies of glucosamine-6-phosphate synthase from Candida albicans. J Mol Biol. 2007;372:672–88. 17681543 10.1016/j.jmb.2007.07.002

[22] Oliveira IA, Allonso D, Fernandes TVA, Lucena DMS, Ventura GT, Dias WB, et al. Enzymatic and structural properties of human glutamine:fructose-6-phosphate amidotransferase 2 (hGFAT2). J Biol Chem. 2021;296:100180. 33303629 10.1074/jbc.RA120.015189 PMC7948480

[23] Richez C, Boetzel J, Floquet N, Koteshwar K, Stevens J, Badet B, et al. Expression and purification of active human internal His(6)-tagged L-glutamine: D-Fructose-6P amidotransferase I. Protein Expr Purif. 2007;54:45–53. 17379537 10.1016/j.pep.2007.01.015

[24] Oki T, Yamazaki K, Kuromitsu J, Okada M, Tanaka I. cDNA cloning and mapping of a novel subtype of glutamine:fructose-6-phosphate amidotransferase (GFAT2) in human and mouse. Genomics. 1999;57:227–34. 10198162 10.1006/geno.1999.5785

[25] Nabeebaccus AA, Verma S, Zoccarato A, Emanuelli G, Santos CX, Streckfuss-Bömeke K, et al. Cardiomyocyte protein O-GlcNAcylation is regulated by GFAT1 not GFAT2. Biochem Biophys Res Commun. 2021;583:121–7. 34735873 10.1016/j.bbrc.2021.10.056 PMC8606754

[26] Chen P, Visokay S, Abrams JM. Drosophila GFAT1 and GFAT2 enzymes encode obligate developmental functions. Fly (Austin). 2020;14:3–9. 32615907 10.1080/19336934.2020.1784674 PMC7714458

[27] Kornfeld R. Studies on L-glutamine D-fructose 6-phosphate amidotransferase. I. Feedback inhibition by uridine diphosphate-N-acetylglucosamine. J Biol Chem. 1967;242:3135–41. 4961641

[28] Broschat KO, Gorka C, Page JD, Martin-Berger CL, Davies MS, Huang Hc H-c, et al. Kinetic characterization of human glutamine-fructose-6-phosphate amidotransferase I: potent feedback inhibition by glucosamine 6-phosphate. J Biol Chem. 2002;277:14764–70. 11842094 10.1074/jbc.M201056200

[29] Li Y, Roux C, Lazereg S, LeCaer J-P, Laprévote O, Badet B, et al. Identification of a novel serine phosphorylation site in human glutamine:fructose-6-phosphate amidotransferase isoform 1. Biochemistry. 2007;46:13163–9. 17941647 10.1021/bi700694c

[30] Moloughney JG, Vega-Cotto NM, Liu S, Patel C, Kim PK, Wu C-C, et al. mTORC2 modulates the amplitude and duration of GFAT1 Ser-243 phosphorylation to maintain flux through the hexosamine pathway during starvation. J Biol Chem. 2018;293:16464–78. 30201609 10.1074/jbc.RA118.003991 PMC6200946

[31] Eguchi S, Oshiro N, Miyamoto T, Yoshino K-I, Okamoto S, Ono T, et al. AMP-activated protein kinase phosphorylates glutamine: fructose-6-phosphate amidotransferase 1 at Ser243 to modulate its enzymatic activity. Genes Cells. 2009;14:179–89. 19170765 10.1111/j.1365-2443.2008.01260.x

[32] Campbell S, Mesaros C, Izzo L, Affronti H, Noji M, Schaffer BE, et al. Glutamine deprivation triggers NAGK-dependent hexosamine salvage. Elife. 2021;10:e62644. 10.7554/eLife.62644 PMC8631944 34844667

[33] Warburg O. On the origin of cancer cells. Science. 1956;123:309–14. 13298683 10.1126/science.123.3191.309

[34] Ge T, Gu X, Jia R, Ge S, Chai P, Zhuang A, et al. Crosstalk between metabolic reprogramming and epigenetics in cancer: updates on mechanisms and therapeutic opportunities. Cancer Commun (Lond). 2022;42:1049–82. 36266736 10.1002/cac2.12374 PMC9648395

[35] Hanahan D. Hallmarks of Cancer: New Dimensions. Cancer Discov. 2022;12:31–46. 35022204 10.1158/2159-8290.CD-21-1059

[36] Siegel RL, Kratzer TB, Giaquinto AN, Sung H, Jemal A. Cancer statistics, 2025. CA Cancer J Clin. 2025;75:10–45. 39817679 10.3322/caac.21871 PMC11745215

[37] Yang C, Peng P, Li L, Shao M, Zhao J, Wang L, et al. High expression of GFAT1 predicts poor prognosis in patients with pancreatic cancer. Sci Rep. 2016;6:39044. 27996048 10.1038/srep39044 PMC5172351

[38] Jia C, Li H, Fu D, Lan Y. GFAT1/HBP/O-GlcNAcylation Axis Regulates β-Catenin Activity to Promote Pancreatic Cancer Aggressiveness. Biomed Res Int. 2020;2020:1921609. 32149084 10.1155/2020/1921609 PMC7048922

[39] Ishino K, Kudo M, Peng W-X, Kure S, Kawahara K, Teduka K, et al. 2-Deoxy-d-glucose increases GFAT1 phosphorylation resulting in endoplasmic reticulum-related apoptosis via disruption of protein N-glycosylation in pancreatic cancer cells. Biochem Biophys Res Commun. 2018;501:668–73. 29753740 10.1016/j.bbrc.2018.05.041

[40] Wang Z, Kuang T, Wu W, Wang D, Lou W, Jin D, et al. GFAT1 is highly expressed in cancer stem cells of pancreatic cancer. Ann Transl Med. 2022;10:544. 35722419 10.21037/atm-22-1946 PMC9201171

[41] Luo G, Jin K, Deng S, Cheng H, Fan Z, Gong Y, et al. Roles of CA19-9 in pancreatic cancer: Biomarker, predictor and promoter. Biochim Biophys Acta Rev Cancer. 2021;1875:188409. 32827580 10.1016/j.bbcan.2020.188409

[42] Singhi AD, Koay EJ, Chari ST, Maitra A. Early Detection of Pancreatic Cancer: Opportunities and Challenges. Gastroenterology. 2019;156:2024–40. 30721664 10.1053/j.gastro.2019.01.259 PMC6486851

[43] Sharma NS, Gupta VK, Garrido VT, Hadad R, Durden BC, Kesh K, et al. Targeting tumor-intrinsic hexosamine biosynthesis sensitizes pancreatic cancer to anti-PD1 therapy. J Clin Invest. 2020;130:451–65. 31613799 10.1172/JCI127515 PMC6934212

[44] Kim PK, Halbrook CJ, Kerk SA, Radyk M, Wisner S, Kremer DM, et al. Hyaluronic acid fuels pancreatic cancer cell growth. Elife. 2021;10:e62645. 10.7554/eLife.62645 PMC8730721 34951587

[45] Hsu Y-S, Wu P-J, Jeng Y-M, Hu C-M, Lee W-H. Differential effects of glucose and N-acetylglucosamine on genome instability. Am J Cancer Res. 2022;12:1556–76. 35530290 PMC9077085

[46] Pennell NA, Arcila ME, Gandara DR, West H. Biomarker Testing for Patients With Advanced Non-Small Cell Lung Cancer: Real-World Issues and Tough Choices. Am Soc Clin Oncol Educ Book. 2019;39:531–42. 10.1200/EDBK_237863 31099633

[47] Chen JW, Dhahbi J. Lung adenocarcinoma and lung squamous cell carcinoma cancer classification, biomarker identification, and gene expression analysis using overlapping feature selection methods. Sci Rep. 2021;11:13323. 34172784 10.1038/s41598-021-92725-8 PMC8233431

[48] Chen W, Do KC, Saxton B, Leng S, Filipczak P, Tessema M, et al. Inhibition of the hexosamine biosynthesis pathway potentiates cisplatin cytotoxicity by decreasing BiP expression in non-small-cell lung cancer cells. Mol Carcinog. 2019;58:1046–55. 30790354 10.1002/mc.22992 PMC6525013

[49] Dragic H, Barthelaix A, Duret C, Le Goupil S, Laprade H, Martin S, et al. The hexosamine pathway and coat complex II promote malignant adaptation to nutrient scarcity. Life Sci Alliance. 2022;5:e202101334. 10.26508/lsa.202101334 PMC9008580 35396334

[50] Wei S, Zhao Q, Zheng K, Liu P, Sha N, Li Y, et al. GFAT1-linked TAB1 glutamylation sustains p38 MAPK activation and promotes lung cancer cell survival under glucose starvation. Cell Discov. 2022;8:77. 35945223 10.1038/s41421-022-00423-0 PMC9363421

[51] Wei S, Ai M, Zhan Y, Yu J, Xie T, Hu Q, et al. TRIM14 suppressed the progression of NSCLC via hexosamine biosynthesis pathway. Carcinogenesis. 2024;45:324–36. 38267812 10.1093/carcin/bgae005

[52] Alisson-Silva F, Freire-de-Lima L, Donadio JL, Lucena MC, Penha L, Sá-Diniz JN, et al. Increase of O-glycosylated oncofetal fibronectin in high glucose-induced epithelial-mesenchymal transition of cultured human epithelial cells. PLoS One. 2013;8:e60471. 23593224 10.1371/journal.pone.0060471 PMC3625189

[53] Taparra K, Wang H, Malek R, Lafargue A, Barbhuiya MA, Wang X, et al. O-GlcNAcylation is required for mutant KRAS-induced lung tumorigenesis. J Clin Invest. 2018;128:4924–37. 30130254 10.1172/JCI94844 PMC6205381

[54] Wang D, Wu J, Wang D, Huang X, Zhang N, Shi Y. Cisplatin enhances protein O‑GlcNAcylation by altering the activity of OGT, OGA and AMPK in human non‑small cell lung cancer cells. Int J Oncol. 2021;58:27. 10.3892/ijo.2021.5207 33846785

[55] Chen W, Saxton B, Tessema M, Belinsky SA. Inhibition of GFAT1 in lung cancer cells destabilizes PD-L1 protein. Carcinogenesis. 2021;42:1171–8. 34270713 10.1093/carcin/bgab063 PMC8491135

[56] Kim J, Lee HM, Cai F, Ko B, Yang C, Lieu EL, et al. The hexosamine biosynthesis pathway is a targetable liability in KRAS/LKB1 mutant lung cancer. Nat Metab. 2020;2:1401–12. 33257855 10.1038/s42255-020-00316-0 PMC7744327

[57] Loibl S, Poortmans P, Morrow M, Denkert C, Curigliano G. Breast cancer. Lancet. 2021;397:1750–69. 33812473 10.1016/S0140-6736(20)32381-3

[58] Xu S, Murtagh S, Han Y, Wan F, Toriola AT. Breast Cancer Incidence Among US Women Aged 20 to 49 Years by Race, Stage, and Hormone Receptor Status. JAMA Netw Open. 2024;7:e2353331. 38277147 10.1001/jamanetworkopen.2023.53331 PMC10818222

[59] Oikari S, Kettunen T, Tiainen S, Häyrinen J, Masarwah A, Sudah M, et al. UDP-sugar accumulation drives hyaluronan synthesis in breast cancer. Matrix Biol. 2018;67:63–74. 29331336 10.1016/j.matbio.2017.12.015

[60] Dong T, Kang X, Liu Z, Zhao S, Ma W, Xuan Q, et al. Altered glycometabolism affects both clinical features and prognosis of triple-negative and neoadjuvant chemotherapy-treated breast cancer. Tumour Biol. 2016;37:8159–68. 26715276 10.1007/s13277-015-4729-8

[61] Chanmee T, Ontong P, Izumikawa T, Higashide M, Mochizuki N, Chokchaitaweesuk C, et al. Hyaluronan Production Regulates Metabolic and Cancer Stem-like Properties of Breast Cancer Cells via Hexosamine Biosynthetic Pathway-coupled HIF-1 Signaling. J Biol Chem. 2016;291:24105–20. 27758869 10.1074/jbc.M116.751263 PMC5104936

[62] Chokchaitaweesuk C, Kobayashi T, Izumikawa T, Itano N. Enhanced hexosamine metabolism drives metabolic and signaling networks involving hyaluronan production and O-GlcNAcylation to exacerbate breast cancer. Cell Death Dis. 2019;10:803. 31645543 10.1038/s41419-019-2034-y PMC6811536

[63] Zhang N, Zhu T, Yu K, Shi M, Wang X, Wang L, et al. Correction to: Elevation of O-GlcNAc and GFAT expression by nicotine exposure promotes epithelial-mesenchymal transition and invasion in breast cancer cells. Cell Death Dis. 2024;15:612. 39174517 10.1038/s41419-024-06900-6 PMC11341762

[64] Ding Q-S, Zhang L, Wang B-C, Zeng Z, Zou X-Q, Cao P-B, et al. Aberrant high expression level of MORC2 is a common character in multiple cancers. Hum Pathol. 2018;76:58–67. 29555576 10.1016/j.humpath.2018.03.011

[65] Pan Z, Ding Q, Guo Q, Guo Y, Wu L, Wu L, et al. MORC2, a novel oncogene, is upregulated in liver cancer and contributes to proliferation, metastasis and chemoresistance. Int J Oncol. 2018;53:59–72. 29620211 10.3892/ijo.2018.4333 PMC5958890

[66] Liu Y-Y, Liu H-Y, Yu T-J, Lu Q, Zhang F-L, Liu G-Y, et al. O-GlcNAcylation of MORC2 at threonine 556 by OGT couples TGF-β signaling to breast cancer progression. Cell Death Differ. 2022;29:861–73. 34974534 10.1038/s41418-021-00901-0 PMC8991186

[67] Torre LA, Bray F, Siegel RL, Ferlay J, Lortet-Tieulent J, Jemal A. Global cancer statistics, 2012. CA Cancer J Clin. 2015;65:87–108. 10.3322/caac.21262 25651787

[68] Li L, Shao M, Peng P, Yang C, Song S, Duan F, et al. High expression of GFAT1 predicts unfavorable prognosis in patients with hepatocellular carcinoma. Oncotarget. 2017;8:19205–17. 28186970 10.18632/oncotarget.15164 PMC5386678

[69] Walter LA, Lin YH, Halbrook CJ, Chuh KN, He L, Pedowitz NJ, et al. Inhibiting the Hexosamine Biosynthetic Pathway Lowers O-GlcNAcylation Levels and Sensitizes Cancer to Environmental Stress. Biochemistry. 2020;59:3169–79. 31625393 10.1021/acs.biochem.9b00560 PMC7231633

[70] Xiang J, Chen C, Liu R, Gou D, Chang L, Deng H, et al. Gluconeogenic enzyme PCK1 deficiency promotes CHK2 O-GlcNAcylation and hepatocellular carcinoma growth upon glucose deprivation. J Clin Invest. 2021;131:e144703. 10.1172/JCI144703 PMC8262473 33690219

[71] Li H, Zhu W, Zhang L, Lei H, Wu X, Guo L, et al. The metabolic responses to hepatitis B virus infection shed new light on pathogenesis and targets for treatment. Sci Rep. 2015;5:8421. 25672227 10.1038/srep08421 PMC4325332

[72] Lin JY, Fisher DE. Melanocyte biology and skin pigmentation. Nature. 2007;445:843–50. 17314970 10.1038/nature05660

[73] Deen AJ, Arasu UT, Pasonen-Seppänen S, Hassinen A, Takabe P, Wojciechowski S, et al. UDP-sugar substrates of HAS3 regulate its O-GlcNAcylation, intracellular traffic, extracellular shedding and correlate with melanoma progression. Cell Mol Life Sci. 2016;73:3183–204. 26883802 10.1007/s00018-016-2158-5 PMC11108457

[74] Jandova J, Wondrak GT. Genomic GLO1 deletion modulates TXNIP expression, glucose metabolism, and redox homeostasis while accelerating human A375 malignant melanoma tumor growth. Redox Biol. 2021;39:101838. 33360689 10.1016/j.redox.2020.101838 PMC7772567

[75] Sobiepanek A, Milner-Krawczyk M, Musolf P, Starecki T, Kobiela T. Anandamide-Modulated Changes in Metabolism, Glycosylation Profile and Migration of Metastatic Melanoma Cells. Cancers (Basel). 2022;14:1419. 10.3390/cancers14061419 PMC8946642 35326572

[76] Duan F, Jia D, Zhao J, Wu W, Min L, Song S, et al. Loss of GFAT1 promotes epithelial-to-mesenchymal transition and predicts unfavorable prognosis in gastric cancer. Oncotarget. 2016;7:38427–39. 27509259 10.18632/oncotarget.9538 PMC5122401

[77] Liu B, Huang Z-B, Chen X, See Y-X, Chen Z-K, Yao H-K. Mammalian Target of Rapamycin 2 (MTOR2) and C-MYC Modulate Glucosamine-6-Phosphate Synthesis in Glioblastoma (GBM) Cells Through Glutamine: Fructose-6-Phosphate Aminotransferase 1 (GFAT1). Cell Mol Neurobiol. 2019;39:415–34. 30771196 10.1007/s10571-019-00659-7 PMC11469801

[78] Zhang Y, Li J, Huang Y, Chen Y, Luo Z, Huang H, et al. Improved antitumor activity against prostate cancer via synergistic targeting of Myc and GFAT-1. Theranostics. 2023;13:578–95. 36632215 10.7150/thno.76614 PMC9830436

[79] Li X, Peng X, Zhang C, Bai X, Li Y, Chen G, et al. Bladder Cancer-Derived Small Extracellular Vesicles Promote Tumor Angiogenesis by Inducing HBP-Related Metabolic Reprogramming and SerRS O-GlcNAcylation in Endothelial Cells. Adv Sci (Weinh). 2022;9:e2202993. 36045101 10.1002/advs.202202993 PMC9596856

[80] Vasconcelos-Dos-Santos A, Loponte HFBR, Mantuano NR, Oliveira IA, de Paula IF, Teixeira LK, et al. Hyperglycemia exacerbates colon cancer malignancy through hexosamine biosynthetic pathway. Oncogenesis. 2017;6:e306. 28319096 10.1038/oncsis.2017.2 PMC5533945

[81] Phoomak C, Vaeteewoottacharn K, Silsirivanit A, Saengboonmee C, Seubwai W, Sawanyawisuth K, et al. High glucose levels boost the aggressiveness of highly metastatic cholangiocarcinoma cells via O-GlcNAcylation. Sci Rep. 2017;7:43842. 28262738 10.1038/srep43842 PMC5338328

[82] Levy D, Larson MG, Vasan RS, Kannel WB, Ho KK. The progression from hypertension to congestive heart failure. JAMA. 1996;275:1557–62. 8622246

[83] Lorell BH, Carabello BA. Left ventricular hypertrophy: pathogenesis, detection, and prognosis. Circulation. 2000;102:470–9. 10908222 10.1161/01.cir.102.4.470

[84] Heineke J, Molkentin JD. Regulation of cardiac hypertrophy by intracellular signalling pathways. Nat Rev Mol Cell Biol. 2006;7:589–600. 16936699 10.1038/nrm1983

[85] Wang ZV, Li DL, Hill JA. Heart failure and loss of metabolic control. J Cardiovasc Pharmacol. 2014;63:302–13. 24336014 10.1097/FJC.0000000000000054 PMC3980079

[86] Chatham JC, Young ME. Metabolic remodeling in the hypertrophic heart: fuel for thought. Circ Res. 2012;111:666–8. 22935530 10.1161/CIRCRESAHA.112.277392 PMC3462817

[87] Ashrafian H, Frenneaux MP, Opie LH. Metabolic mechanisms in heart failure. Circulation. 2007;116:434–48. 17646594 10.1161/CIRCULATIONAHA.107.702795

[88] Gibb AA, Hill BG. Metabolic Coordination of Physiological and Pathological Cardiac Remodeling. Circ Res. 2018;123:107–28. 29929976 10.1161/CIRCRESAHA.118.312017 PMC6023588

[89] Tran DH, Wang ZV. Glucose Metabolism in Cardiac Hypertrophy and Heart Failure. J Am Heart Assoc. 2019;8:e012673. 31185774 10.1161/JAHA.119.012673 PMC6645632

[90] Ardehali H, Sabbah HN, Burke MA, Sarma S, Liu PP, Cleland JGF, et al. Targeting myocardial substrate metabolism in heart failure: potential for new therapies. Eur J Heart Fail. 2012;14:120–9. 22253453 10.1093/eurjhf/hfr173 PMC3260022

[91] Lopaschuk GD, Ussher JR, Folmes CDL, Jaswal JS, Stanley WC. Myocardial fatty acid metabolism in health and disease. Physiol Rev. 2010;90:207–58. 20086077 10.1152/physrev.00015.2009

[92] Leong HS, Brownsey RW, Kulpa JE, Allard MF. Glycolysis and pyruvate oxidation in cardiac hypertrophy–why so unbalanced? Comp Biochem Physiol A Mol Integr Physiol. 2003;135:499–513. 12890541 10.1016/s1095-6433(03)00007-2

[93] Young ME, Yan J, Razeghi P, Cooksey RC, Guthrie PH, Stepkowski SM, et al. Proposed regulation of gene expression by glucose in rodent heart. Gene Regul Syst Bio. 2007;1:251–62. 19936093 10.4137/grsb.s222 PMC2759127

[94] Lunde IG, Aronsen JM, Kvaløy H, Qvigstad E, Sjaastad I, Tønnessen T, et al. Cardiac O-GlcNAc signaling is increased in hypertrophy and heart failure. Physiol Genomics. 2012;44:162–72. 22128088 10.1152/physiolgenomics.00016.2011

[95] van Berlo JH, Maillet M, Molkentin JD. Signaling effectors underlying pathologic growth and remodeling of the heart. J Clin Invest. 2013;123:37–45. 23281408 10.1172/JCI62839 PMC3533272

[96] Tran DH, May HI, Li Q, Luo X, Huang J, Zhang G, et al. Chronic activation of hexosamine biosynthesis in the heart triggers pathological cardiac remodeling. Nat Commun. 2020;11:1771. 32286306 10.1038/s41467-020-15640-y PMC7156663

[97] Ishikita A, Matsushima S, Ikeda S, Okabe K, Nishimura R, Tadokoro T, et al. GFAT2 mediates cardiac hypertrophy through HBP-O-GlcNAcylation-Akt pathway. iScience. 2021;24:103517. 34934932 10.1016/j.isci.2021.103517 PMC8661546

[98] Gélinas R, Mailleux F, Dontaine J, Bultot L, Demeulder B, Ginion A, et al. AMPK activation counteracts cardiac hypertrophy by reducing O-GlcNAcylation. Nat Commun. 2018;9:374. 29371602 10.1038/s41467-017-02795-4 PMC5785516

[99] Zhu WZ, Ledee D, Olson AK. Temporal regulation of protein O-GlcNAc levels during pressure-overload cardiac hypertrophy. Physiol Rep. 2021;9:e14965. 34337900 10.14814/phy2.14965 PMC8326887

[100] Maya L, Villarreal FJ. Diagnostic approaches for diabetic cardiomyopathy and myocardial fibrosis. J Mol Cell Cardiol. 2010;48:524–9. 19595694 10.1016/j.yjmcc.2009.06.021 PMC2824060

[101] Boudina S, Abel ED. Diabetic cardiomyopathy revisited. Circulation. 2007;115:3213–23. 17592090 10.1161/CIRCULATIONAHA.106.679597

[102] Fricovsky ES, Suarez J, Ihm S-H, Scott BT, Suarez-Ramirez JA, Banerjee I, et al. Excess protein O-GlcNAcylation and the progression of diabetic cardiomyopathy. Am J Physiol Regul Integr Comp Physiol. 2012;303:R689–R99. 22874425 10.1152/ajpregu.00548.2011 PMC3469670

[103] De Blasio MJ, Huynh N, Deo M, Dubrana LE, Walsh J, Willis A, et al. Defining the Progression of Diabetic Cardiomyopathy in a Mouse Model of Type 1 Diabetes. Front Physiol. 2020;11:124. 32153425 10.3389/fphys.2020.00124 PMC7045054

[104] Buse MG. Hexosamines, insulin resistance, and the complications of diabetes: current status. Am J Physiol Endocrinol Metab. 2006;290:E1–E8. 16339923 10.1152/ajpendo.00329.2005 PMC1343508

[105] Clark RJ, McDonough PM, Swanson E, Trost SU, Suzuki M, Fukuda M, et al. Diabetes and the accompanying hyperglycemia impairs cardiomyocyte calcium cycling through increased nuclear O-GlcNAcylation. J Biol Chem. 2003;278:44230–7. 12941958 10.1074/jbc.M303810200

[106] Ziyadeh FN, Sharma K, Ericksen M, Wolf G. Stimulation of collagen gene expression and protein synthesis in murine mesangial cells by high glucose is mediated by autocrine activation of transforming growth factor-beta. J Clin Invest. 1994;93:536–42. 8113392 10.1172/JCI117004 PMC293875

[107] Jensen RV, Zachara NE, Nielsen PH, Kimose HH, Kristiansen SB, Bøtker HE. Impact of O-GlcNAc on cardioprotection by remote ischaemic preconditioning in non-diabetic and diabetic patients. Cardiovasc Res. 2013;97:369–78. 23201773 10.1093/cvr/cvs337 PMC3584969

[108] Gilbert RE, Connelly K, Kelly DJ, Pollock CA, Krum H. Heart failure and nephropathy: catastrophic and interrelated complications of diabetes. Clin J Am Soc Nephrol. 2006;1:193–208. 17699208 10.2215/CJN.00540705

[109] Erickson JR, Pereira L, Wang L, Han G, Ferguson A, Dao K, et al. Diabetic hyperglycaemia activates CaMKII and arrhythmias by O-linked glycosylation. Nature. 2013;502:372–6. 24077098 10.1038/nature12537 PMC3801227

[110] Dubois-Deruy E, Belliard A, Mulder P, Bouvet M, Smet-Nocca C, Janel S, et al. Interplay between troponin T phosphorylation and O-N-acetylglucosaminylation in ischaemic heart failure. Cardiovasc Res. 2015;107:56–65. 25916824 10.1093/cvr/cvv136

[111] Kim Y-H, Song M, Oh Y-S, Heo K, Choi J-W, Park J-M, et al. Inhibition of phospholipase C-beta1-mediated signaling by O-GlcNAc modification. J Cell Physiol. 2006;207:689–96. 16538662 10.1002/jcp.20609

[112] Zhu-Mauldin X, Marsh SA, Zou L, Marchase RB, Chatham JC. Modification of STIM1 by O-linked N-acetylglucosamine (O-GlcNAc) attenuates store-operated calcium entry in neonatal cardiomyocytes. J Biol Chem. 2012;287:39094–106. 22992728 10.1074/jbc.M112.383778 PMC3493950

[113] Nagy T, Champattanachai V, Marchase RB, Chatham JC. Glucosamine inhibits angiotensin II-induced cytoplasmic Ca2+ elevation in neonatal cardiomyocytes via protein-associated O-linked N-acetylglucosamine. Am J Physiol Cell Physiol. 2005;290:C57–C65. 16107505 10.1152/ajpcell.00263.2005

[114] Chatham JC, Young ME, Zhang J. Role of O-linked N-acetylglucosamine (O-GlcNAc) modification of proteins in diabetic cardiovascular complications. Curr Opin Pharmacol. 2021;57:1–12. 10.1016/j.coph.2020.08.005 PMC9027139 32937226

[115] Sack MN, Disch DL, Rockman HA, Kelly DP. A role for Sp and nuclear receptor transcription factors in a cardiac hypertrophic growth program. Proc Natl Acad Sci USA. 1997;94:6438–43. 9177236 10.1073/pnas.94.12.6438 PMC21068

[116] Hulot J-S, Fauconnier J, Ramanujam D, Chaanine A, Aubart F, Sassi Y, et al. Critical role for stromal interaction molecule 1 in cardiac hypertrophy. Circulation. 2011;124:796–805. 21810664 10.1161/CIRCULATIONAHA.111.031229 PMC3428713

[117] Muthusamy S, DeMartino AM, Watson LJ, Brittian KR, Zafir A, Dassanayaka S, et al. MicroRNA-539 is up-regulated in failing heart, and suppresses O-GlcNAcase expression. J Biol Chem. 2014;289:29665–76. 25183011 10.1074/jbc.M114.578682 PMC4207981

[118] Weinberg JM. The cell biology of ischemic renal injury. Kidney Int. 1991;39:476–500. 2062034 10.1038/ki.1991.58

[119] Zimmerman BJ, Granger DN. Mechanisms of reperfusion injury. Am J Med Sci. 1994;307:284–92. 8160724 10.1097/00000441-199404000-00009

[120] Shoskes DA, Halloran PF. Delayed graft function in renal transplantation: etiology, management and long-term significance. J Urol. 1996;155:1831–40. 8618268 10.1016/s0022-5347(01)66023-3

[121] Reilly PM, Schiller HJ, Bulkley GB. Pharmacologic approach to tissue injury mediated by free radicals and other reactive oxygen metabolites. Am J Surg. 1991;161:488–503. 2035771 10.1016/0002-9610(91)91120-8

[122] Weight SC, Bell PR, Nicholson ML. Renal ischaemia–reperfusion injury. Br J Surg. 1996;83:162–70. 8689154

[123] Land W, Messmer K, Events E. The Impact of Ischemia/Reperfusion Injury on Specific and Non-Specific, Early and Late Chronic Events After Organ Transplantation. Transplantation Reviews. 1996;10:108–27.

[124] Granger DN, Korthuis RJ. Physiologic mechanisms of postischemic tissue injury. Annu Rev Physiol. 1995;57:311–32. 7778871 10.1146/annurev.ph.57.030195.001523

[125] Wang ZV, Deng Y, Gao N, Pedrozo Z, Li DL, Morales CR, et al. Spliced X-box binding protein 1 couples the unfolded protein response to hexosamine biosynthetic pathway. Cell. 2014;156:1179–92. 24630721 10.1016/j.cell.2014.01.014 PMC3959665

[126] Liu J, Marchase RB, Chatham JC. Glutamine-induced protection of isolated rat heart from ischemia/reperfusion injury is mediated via the hexosamine biosynthesis pathway and increased protein O-GlcNAc levels. J Mol Cell Cardiol. 2007;42:177–85. 17069847 10.1016/j.yjmcc.2006.09.015 PMC1779903

[127] Raman P, Krukovets I, Marinic TE, Bornstein P, Stenina OI. Glycosylation mediates up-regulation of a potent antiangiogenic and proatherogenic protein, thrombospondin-1, by glucose in vascular smooth muscle cells. J Biol Chem. 2007;282:5704–14. 17178709 10.1074/jbc.M610965200

[128] Sage AT, Walter LA, Shi Y, Khan MI, Kaneto H, Capretta A, et al. Hexosamine biosynthesis pathway flux promotes endoplasmic reticulum stress, lipid accumulation, and inflammatory gene expression in hepatic cells. Am J Physiol Endocrinol Metab. 2010;298:E499–E511. 19952345 10.1152/ajpendo.00507.2009

[129] DeFronzo RA, Ferrannini E, Groop L, Henry RR, Herman WH, Holst JJ, et al. Type 2 diabetes mellitus. Nat Rev Dis Primers. 2015;1:15019. 27189025 10.1038/nrdp.2015.19

[130] Chou K-C. Molecular therapeutic target for type-2 diabetes. J Proteome Res. 2004;3:1284–8. 15595739 10.1021/pr049849v

[131] Hebert LF, Daniels MC, Zhou J, Crook ED, Turner RL, Simmons ST, et al. Overexpression of glutamine:fructose-6-phosphate amidotransferase in transgenic mice leads to insulin resistance. J Clin Invest. 1996;98:930–6. 8770864 10.1172/JCI118876 PMC507507

[132] Marshall S, Bacote V, Traxinger RR. Complete inhibition of glucose-induced desensitization of the glucose transport system by inhibitors of mRNA synthesis. Evidence for rapid turnover of glutamine:fructose-6-phosphate amidotransferase. J Biol Chem. 1991;266:10155–61. 2037572

[133] Buse MG, Robinson KA, Marshall BA, Mueckler M. Differential effects of GLUT1 or GLUT4 overexpression on hexosamine biosynthesis by muscles of transgenic mice. J Biol Chem. 1996;271:23197–202. 8798515 10.1074/jbc.271.38.23197

[134] Bhonagiri P, Pattar GR, Habegger KM, McCarthy AM, Tackett L, Elmendorf JS. Evidence coupling increased hexosamine biosynthesis pathway activity to membrane cholesterol toxicity and cortical filamentous actin derangement contributing to cellular insulin resistance. Endocrinology. 2011;152:3373–84. 21712361 10.1210/en.2011-1295 PMC3159786

[135] Covert JD, Grice BA, Thornburg MG, Kaur M, Ryan AP, Tackett L, et al. An early, reversible cholesterolgenic etiology of diet-induced insulin resistance. Mol Metab. 2023;72:101715. 37019209 10.1016/j.molmet.2023.101715 PMC10114231

[136] Kaneto H, Xu G, Song KH, Suzuma K, Bonner-Weir S, Sharma A, et al. Activation of the hexosamine pathway leads to deterioration of pancreatic beta-cell function through the induction of oxidative stress. J Biol Chem. 2001;276:31099–104. 11390407 10.1074/jbc.M104115200

[137] Zhang W, Liu J, Tian L, Liu Q, Fu Y, Garvey WT. TRIB3 mediates glucose-induced insulin resistance via a mechanism that requires the hexosamine biosynthetic pathway. Diabetes. 2013;62:4192–200. 23990361 10.2337/db13-0312 PMC3837074

[138] Bosch RR, Pouwels M-JJM, Span PN, Olthaar AJ, Tack CJ, Hermus ARMM, et al. Hexosamines are unlikely to function as a nutrient-sensor in 3T3-L1 adipocytes: a comparison of UDP-hexosamine levels after increased glucose flux and glucosamine treatment. Endocrine. 2004;23:17–24. 15034192 10.1385/endo:23:1:17

[139] Nathan DM, Genuth S, Lachin J, Cleary P, Crofford O, Davis M, et al. The effect of intensive treatment of diabetes on the development and progression of long-term complications in insulin-dependent diabetes mellitus. N Engl J Med. 1993;329:977–86. 8366922 10.1056/NEJM199309303291401

[140] Lorenzi M. Glucose toxicity in the vascular complications of diabetes: the cellular perspective. Diabetes Metab Rev. 1992;8:85–103. 10.1002/dmr.5610080202 1425126

[141] Sayeski PP, Kudlow JE. Glucose Metabolism to Glucosamine Is Necessary for Glucose Stimulation of Transforming Growth Factor-α Gene Transcription*. Journal of Biological Chemistry. 1996;271:15237–43. 8663078 10.1074/jbc.271.25.15237

[142] Mueller SG, Paterson AJ, Kudlow JE. Transforming growth factor alpha in arterioles: cell surface processing of its precursor by elastases. Mol Cell Biol. 1990;10:4596–602. 2201895 10.1128/mcb.10.9.4596-4602.1990 PMC361048

[143] Klagsbrun M, Edelman ER. Biological and biochemical properties of fibroblast growth factors. Implications for the pathogenesis of atherosclerosis. Arteriosclerosis. 1989;9:269–78. 2655570 10.1161/01.atv.9.3.269

[144] Lindner V, Lappi DA, Baird A, Majack RA, Reidy MA. Role of basic fibroblast growth factor in vascular lesion formation. Circ Res. 1991;68:106–13. 1984855 10.1161/01.res.68.1.106

[145] Sayeski PP, Kudlow JE. Glucose metabolism to glucosamine is necessary for glucose stimulation of transforming growth factor-alpha gene transcription. J Biol Chem. 1996;271:15237–43. 8663078 10.1074/jbc.271.25.15237

[146] Dong W, Imdad L, Xu S, Wang Y, Liu C, Song S, et al. O-GlcNAc Modification Is a Promising Therapeutic Target for Diabetic Retinopathy. Int J Mol Sci. 2024;25:6286. 10.3390/ijms25116286 PMC11173153 38892474

[147] Dai W, Dierschke SK, Toro AL, Dennis MD. Consumption of a high fat diet promotes protein O-GlcNAcylation in mouse retina via NR4A1-dependent GFAT2 expression. Biochim Biophys Acta Mol Basis Dis. 2018;1864:3568–76. 30254013 10.1016/j.bbadis.2018.09.006 PMC6239931

[148] Chatterjee A, Eshwaran R, Poschet G, Lomada S, Halawa M, Wilhelm K, et al. Involvement of NDPK-B in Glucose Metabolism-Mediated Endothelial Damage via Activation of the Hexosamine Biosynthesis Pathway and Suppression of O-GlcNAcase Activity. Cells. 2020;9:2324. 10.3390/cells9102324 PMC7588982 33086728

[149] Locatelli F, Canaud B, Eckardt K-U, Stenvinkel P, Wanner C, Zoccali C. The importance of diabetic nephropathy in current nephrological practice. Nephrol Dial Transplant. 2003;18:1716–25. 12937216 10.1093/ndt/gfg288

[150] Schleicher ED, Weigert C. Role of the hexosamine biosynthetic pathway in diabetic nephropathy. Kidney Int Suppl. 2000;77:S13–S8. 10997685 10.1046/j.1523-1755.2000.07703.x

[151] James LR, Ingram A, Ly H, Thai K, Cai L, Scholey JW. Angiotensin II activates the GFAT promoter in mesangial cells. Am J Physiol Renal Physiol. 2001;281:F151–F62. 11399656 10.1152/ajprenal.2001.281.1.F151

[152] Weigert C, Brodbeck K, Sawadogo M, Häring HU, Schleicher ED. Upstream stimulatory factor (USF) proteins induce human TGF-beta1 gene activation via the glucose-response element-1013/-1002 in mesangial cells: up-regulation of USF activity by the hexosamine biosynthetic pathway. J Biol Chem. 2004;279:15908–15. 14757763 10.1074/jbc.M313524200

[153] Kolm-Litty V, Sauer U, Nerlich A, Lehmann R, Schleicher ED. High glucose-induced transforming growth factor beta1 production is mediated by the hexosamine pathway in porcine glomerular mesangial cells. J Clin Invest. 1998;101:160–9. 9421478 10.1172/JCI119875 PMC508552

[154] Weigert C, Brodbeck K, Lehmann R, Häring HU, Schleicher ED. Overexpression of glutamine:fructose-6-phosphate-amidotransferase induces transforming growth factor-beta1 synthesis in NIH-3T3 fibroblasts. FEBS Lett. 2001;488:95–9. 11163803 10.1016/s0014-5793(00)02395-4

[155] James LR, Fantus IG, Goldberg H, Ly H, Scholey JW. Overexpression of GFAT activates PAI-1 promoter in mesangial cells. Am J Physiol Renal Physiol. 2000;279:F718–F27. 10997922 10.1152/ajprenal.2000.279.4.F718

[156] James LR, Le C, Scholey JW. Influence of glucosamine on glomerular mesangial cell turnover: implications for hyperglycemia and hexosamine pathway flux. Am J Physiol Endocrinol Metab. 2010;298:E210–E21. 19903862 10.1152/ajpendo.00232.2009 PMC2822474

[157] Nerlich AG, Sauer U, Kolm-Litty V, Wagner E, Koch M, Schleicher ED. Expression of glutamine:fructose-6-phosphate amidotransferase in human tissues: evidence for high variability and distinct regulation in diabetes. Diabetes. 1998;47:170–8. 9519709 10.2337/diab.47.2.170

[158] Jaiswal YS, Tatke PA, Gabhe SY, Vaidya AB. Antidiabetic activity of extracts of Anacardium occidentale Linn. leaves on n-streptozotocin diabetic rats. J Tradit Complement Med. 2016;7:421–7. 29034189 10.1016/j.jtcme.2016.11.007 PMC5634720

[159] Ukwenya VO, Adelakun SA, Elekofehinti OO. Exploring the antidiabetic potential of compounds isolated from Anacardium occidentale using computational aproach: ligand-based virtual screening. In Silico Pharmacol. 2021;9:25. 33868895 10.1007/s40203-021-00084-z PMC8019409

[160] Gull H, Ikram A, Khalil AA, Ahmed Z, Nemat A. Assessing the multitargeted antidiabetic potential of three pomegranate peel-specific metabolites: An in silico and pharmacokinetics study. Food Sci Nutr. 2023;11:7188–205. 37970376 10.1002/fsn3.3644 PMC10630828

[161] Ashraf SA, Elkhalifa AEO, Mehmood K, Adnan M, Khan MA, Eltoum NE, et al. Multi-Targeted Molecular Docking, Pharmacokinetics, and Drug-Likeness Evaluation of Okra-Derived Ligand Abscisic Acid Targeting Signaling Proteins Involved in the Development of Diabetes. Molecules. 2021;26:5957. 10.3390/molecules26195957 PMC8512114 34641501

[162] Damián-Medina K, Salinas-Moreno Y, Milenkovic D, Figueroa-Yáñez L, Marino-Marmolejo E, Higuera-Ciapara I, et al. In silico analysis of antidiabetic potential of phenolic compounds from blue corn (Zea mays L.) and black bean (Phaseolus vulgaris L.). Heliyon. 2020;6:e03632. 32258479 10.1016/j.heliyon.2020.e03632 PMC7110303

[163] Derakhshanian H, Djalali M, Mohammad Hassan MH, Alvandi E, Eshraghian MR, Mirshafiey A, et al. Vitamin D suppresses cellular pathways of diabetes complication in liver. Iran J Basic Med Sci. 2019;22:690–4. 31231498 10.22038/ijbms.2019.36054.8584 PMC6570757

[164] Derakhshanian H, Djazayery A, Javanbakht MH, Eshraghian MR, Mirshafiey A, Zarei M, et al. The Effect of Vitamin D on Cellular Pathways of Diabetic Nephropathy. Rep Biochem Mol Biol. 2019;7:217–22. 30805403 PMC6374056

[165] Jia ZH, Liu ZH, Zheng JM, Zeng CH, Li LS. Combined therapy of rhein and benazepril on the treatment of diabetic nephropathy in db/db mice. Exp Clin Endocrinol Diabetes. 2007;115:571–6. 17943690 10.1055/s-2007-981469

[166] Zheng JM, Zhu JM, Li LS, Liu ZH. Rhein reverses the diabetic phenotype of mesangial cells over-expressing the glucose transporter (GLUT1) by inhibiting the hexosamine pathway. Br J Pharmacol. 2008;153:1456–64. 18264122 10.1038/bjp.2008.26 PMC2437903

[167] Zuliani I, Lanzillotta C, Tramutola A, Barone E, Perluigi M, Rinaldo S, et al. High-Fat Diet Leads to Reduced Protein O-GlcNAcylation and Mitochondrial Defects Promoting the Development of Alzheimer’s Disease Signatures. Int J Mol Sci. 2021;22:3746. 10.3390/ijms22073746 PMC8038495 33916835

[168] Liu F, Shi J, Tanimukai H, Gu J, Gu J, Grundke-Iqbal I, et al. Reduced O-GlcNAcylation links lower brain glucose metabolism and tau pathology in Alzheimer’s disease. Brain. 2009;132:1820–32. 19451179 10.1093/brain/awp099 PMC2702834

[169] Jackson SP, Tjian R. O-glycosylation of eukaryotic transcription factors: implications for mechanisms of transcriptional regulation. Cell. 1988;55:125–33. 3139301 10.1016/0092-8674(88)90015-3

[170] Yki-Järvinen H, Virkamäki A, Daniels MC, McClain D, Gottschalk WK. Insulin and glucosamine infusions increase O-linked N-acetyl-glucosamine in skeletal muscle proteins in vivo. Metabolism. 1998;47:449–55. 9550544 10.1016/s0026-0495(98)90058-0

[171] Zhang Z, Li X, Guo W, Huang Z. Enhancing GFPT1 expression with glutamine protects chondrocytes in osteoarthritis. Int Immunopharmacol. 2024;143:113427. 39426230 10.1016/j.intimp.2024.113427

[172] Kelly GS. The role of glucosamine sulfate and chondroitin sulfates in the treatment of degenerative joint disease. Altern Med Rev. 1998;3:27–39. 9600024

[173] Reichelt A, Förster KK, Fischer M, Rovati LC, Setnikar I. Efficacy and safety of intramuscular glucosamine sulfate in osteoarthritis of the knee. A randomised, placebo-controlled, double-blind study. Arzneimittelforschung. 1994;44:75–80. 8135881

[174] Gouze JN, Gouze E, Palmer GD, Kaneto H, Ghivizzani SC, Grodzinsky AJ, et al. Adenovirus-mediated gene transfer of glutamine: fructose-6-phosphate amidotransferase antagonizes the effects of interleukin-1beta on rat chondrocytes. Osteoarthritis Cartilage. 2004;12:217–24. 14972338 10.1016/j.joca.2003.11.002

[175] Thomas AC, Hubbard-Turner T, Wikstrom EA, Palmieri-Smith RM. Epidemiology of Posttraumatic Osteoarthritis. Journal of Athletic Training. 2017;52:491–6. 27145096 10.4085/1062-6050-51.5.08 PMC5488839

[176] Riegger J, Baumert J, Zaucke F, Brenner RE. The Hexosamine Biosynthetic Pathway as a Therapeutic Target after Cartilage Trauma: Modification of Chondrocyte Survival and Metabolism by Glucosamine Derivatives and PUGNAc in an Ex Vivo Model. Int J Mol Sci. 2021;22:7247. 10.3390/ijms22147247 PMC8305151 34298867

[177] Jagannath S, Mallanna SH, Nandini CD. Diet-inducing hypercholesterolemia show decreased O-GlcNAcylation of liver proteins through modulation of AMPK. J Physiol Biochem. 2023;80:205–18. 37996652 10.1007/s13105-023-00997-7

[178] Moloughney JG, Kim PK, Vega-Cotto NM, Wu C-C, Zhang S, Adlam M, et al. mTORC2 Responds to Glutamine Catabolite Levels to Modulate the Hexosamine Biosynthesis Enzyme GFAT1. Mol Cell. 2016;63:811–26. 27570073 10.1016/j.molcel.2016.07.015 PMC5006067

[179] Wu K, Chen L, Qiu Z, Zhao B, Hou J, Lei S, et al. Protective Effect and Mechanism of Xbp1s Regulating HBP/O-GlcNAcylation through GFAT1 on Brain Injury after SAH. Biomedicines. 2023;11:1259. 10.3390/biomedicines11051259 PMC10215646 37238930

[180] Görg B, Karababa A, Schütz E, Paluschinski M, Schrimpf A, Shafigullina A, et al. O-GlcNAcylation-dependent upregulation of HO1 triggers ammonia-induced oxidative stress and senescence in hepatic encephalopathy. J Hepatol. 2019;71:930–41. 31279900 10.1016/j.jhep.2019.06.020

[181] Silva-Aguiar RP, Bezerra NCF, Lucena MC, Sirtoli GM, Sudo RT, Zapata-Sudo G, et al. O-GlcNAcylation reduces proximal tubule protein reabsorption and promotes proteinuria in spontaneously hypertensive rats. J Biol Chem. 2018;293:12749–58. 29954945 10.1074/jbc.RA118.001746 PMC6102134

[182] Thenappan T, Shah SJ, Rich S, Tian L, Archer SL, Gomberg-Maitland M. Survival in pulmonary arterial hypertension: a reappraisal of the NIH risk stratification equation. Eur Respir J. 2010;35:1079–87. 20032020 10.1183/09031936.00072709 PMC8782564

[183] Benza RL, Miller DP, Gomberg-Maitland M, Frantz RP, Foreman AJ, Coffey CS, et al. Predicting survival in pulmonary arterial hypertension: insights from the Registry to Evaluate Early and Long-Term Pulmonary Arterial Hypertension Disease Management (REVEAL). Circulation. 2010;122:164–72. 20585012 10.1161/CIRCULATIONAHA.109.898122

[184] Humbert M, Sitbon O, Yaïci A, Montani D, O’Callaghan DS, Jaïs X, et al. Survival in incident and prevalent cohorts of patients with pulmonary arterial hypertension. Eur Respir J. 2010;36:549–55. 20562126 10.1183/09031936.00057010

[185] Prisco SZ, Rose L, Potus F, Tian L, Wu D, Hartweck L, et al. Excess Protein O-GlcNAcylation Links Metabolic Derangements to Right Ventricular Dysfunction in Pulmonary Arterial Hypertension. Int J Mol Sci. 2020;21:7278. 10.3390/ijms21197278 PMC7582480 33019763

[186] Bharadwaj S, Singh M, Kirtipal N, Kang SG. SARS-CoV-2 and Glutamine: SARS-CoV-2 Triggered Pathogenesis via Metabolic Reprograming of Glutamine in Host Cells. Front Mol Biosci. 2020;7:627842. 33585567 10.3389/fmolb.2020.627842 PMC7873863

[187] Naganuma A, Furuchi T, Miura N, Hwang G-W, Kuge S. Investigation of intracellular factors involved in methylmercury toxicity. Tohoku J Exp Med. 2002;196:65–70. 12498317 10.1620/tjem.196.65

[188] Miura N, Kaneko S, Hosoya S, Furuchi T, Miura K, Kuge S, et al. Overexpression of L-glutamine:D-fructose-6-phosphate amidotransferase provides resistance to methylmercury in Saccharomyces cerevisiae. FEBS Lett. 1999;458:215–8. 10481068 10.1016/s0014-5793(99)01158-8

[189] Asthana A, Ramakrishnan P, Vicioso Y, Zhang K, Parameswaran R. Hexosamine Biosynthetic Pathway Inhibition Leads to AML Cell Differentiation and Cell Death. Mol Cancer Ther. 2018;17:2226–37. 30082471 10.1158/1535-7163.MCT-18-0426 PMC6168390

[190] Kim S-M, Zhang S, Park J, Sung HJ, Tran T-DT, Chung C, et al. REM Sleep Deprivation Impairs Learning and Memory by Decreasing Brain O-GlcNAc Cycling in Mouse. Neurotherapeutics. 2021;18:2504–17. 34312767 10.1007/s13311-021-01094-7 PMC8804064

